# Genome-Wide Analysis of Sex Disparities in the Genetic Architecture of Lung and Colorectal Cancers

**DOI:** 10.3390/genes12050686

**Published:** 2021-05-01

**Authors:** Alireza Nazarian, Alexander M. Kulminski

**Affiliations:** Biodemography of Aging Research Unit, Social Science Research Institute, Duke University, Durham, NC 27705, USA; alireza.nazarian@duke.edu

**Keywords:** genetic heterogeneity, sex-specific genetic polymorphisms, GWAS, CRCa, LCa

## Abstract

Almost all complex disorders have manifested epidemiological and clinical sex disparities which might partially arise from sex-specific genetic mechanisms. Addressing such differences can be important from a precision medicine perspective which aims to make medical interventions more personalized and effective. We investigated sex-specific genetic associations with colorectal (CRCa) and lung (LCa) cancers using genome-wide single-nucleotide polymorphisms (SNPs) data from three independent datasets. The genome-wide association analyses revealed that 33 SNPs were associated with CRCa/LCa at *P* < 5.0 × 10^−6^ neither males or females. Of these, 26 SNPs had sex-specific effects as their effect sizes were statistically different between the two sexes at a Bonferroni-adjusted significance level of 0.0015. None had proxy SNPs within their ±1 Mb regions and the closest genes to 32 SNPs were not previously associated with the corresponding cancers. The pathway enrichment analyses demonstrated the associations of 35 pathways with CRCa or LCa which were mostly implicated in immune system responses, cell cycle, and chromosome stability. The significant pathways were mostly enriched in either males or females. Our findings provided novel insights into the potential sex-specific genetic heterogeneity of CRCa and LCa at SNP and pathway levels.

## 1. Introduction

Sex disparities have been long reported in various malignancies, with most cancers predominantly affecting males and having better survival and lower mortality rates in females [1,2,3,4,5]. Lung (LCa) and colorectal (CRCa) cancers are among the top three common malignancies in both males and females. They jointly comprised around 25.1% of new cancer cases in men and 17.6% in women in 2018. They were also among the leading causes of cancer-related deaths in 2018, accounting for around 31% and 23% of such deaths in males and females [6]. A study of the relative risks of different cancer types revealed that LCa and CRCa were among 32 other cancers that had significantly higher incidence rates in men across various geographical regions and gross domestic product (GDP) groups, with average male-to-female incidence rate ratios of 2.08 and 1.33, respectively [3]. Sex has also been suggested as a potential favorable prognostic factor for these cancers conferring better survival to female patients [2,7,8,9]. LCa and CRCa were reported to have male-to-female mortality ratios of 2.31 and 1.42, respectively, and worse survival in males with significant male-to-female hazards ratios of 1.17 and 1.08 after adjusting models for the age of subjects and stage of tumors [2]. 

In addition to the sex-dependent differences in incidence, survival, and mortality rates, LCa and CRCa have displayed some other clinical and histopathological sex disparities in tumor topology, clinical manifestations, aggression potentials, and responses to therapy [10,11,12]. For instance, several studies reported that the female-to-male ratios were >1 and <1 in right- and left-sided CRCa, respectively, which have different clinical manifestations and are genetically heterogeneous [12,13,14]. As another example, while adenocarcinoma was found to be the most common LCa subtype in both sexes in most populations, the proportion of adenocarcinomas to squamous cell carcinomas was different in the two sexes [15]. It was also suggested that women might be more susceptible than men to tobacco carcinogenesis [11]. Sex disparities have been reported in the metastasis patterns of LCa and CRCa as well. The major distant metastatic sites of LCa include brain, bone, liver, and adrenal glands; and CRCa mainly metastasizes into the liver, lung, and bone [16]. It was suggested that bone metastases from LCa affects females more frequently than males possibly due to a feminized bone microenvironment [17], however, LCa-related brain metastases mostly occur in male patients [18]. Additionally, while the proportions of advanced-stage (i.e., Duke C and D) right- and left-sided CRCa were not statistically different in females, male patients with right-sided CRCa were more likely to present at advanced-stages compared to those with left-sided tumors [19]. Another aspect of sex differences was highlighted in previous reports indicating that female patients better responded to surgical treatment and chemotherapy of non-small cell LCa and CRCa than male patients [4]. In addition, females were found more susceptible than males to develop chemotherapy-related side effects (e.g., stomatitis, hematologic, and gastrointestinal toxicity) [8,20].

These disparities tend to be attributed to different life expectancies of the two sexes, hormonal effects, environmental exposures, and lifestyle risk factors. In addition, they might be in part mediated by the genetic and epigenetic mechanisms [5,10,21,22]. LCa and CRCa have evident genetic bases and in most cases are caused sporadically as multi-factorial disorders [23]. The genomes of patients with LCa and microsatellite stable CRCa may harbor ~10 to ~200 somatic mutations, mostly single-base substitutions, and CRCa cases with microsatellite instability (MSI) may accumulate >500 somatic mutations in their genomes [24]. The mutation density in the genomes of cancer patients demonstrated sex disparities as the numbers of single nucleotide mutations were in general higher in males than female [4]. It has been reported that LCa-linked mutations in *P53*, *K-RAS*, and *EGFR* genes are more commonly found in women than in men [11]. Additionally, while *P53*, *APC*, and *K-RAS* mutations are more commonly found in left-sided CRCa (more common in males), MSI, *BRAF* mutations, and *eNOS* and *EPHB4* overexpression are more common in right-sided tumors (more common in females) [12,25]. More prevalent MSI-high (MSI-H) tumors in women with right-sided CRCa may confer them a decreased metastasis propensity [19]. In addition, genome-wide association studies (GWAS) have discovered several sex-dependent LCa/CRCa-associated single-nucleotide polymorphisms (SNPs) and haplotypes [22,26,27].

Genetic factors were also suggested to moderate the gender-specific recurrence and survival rates [10,12,28,29,30,31]. For instance, in a study of stage-III CRCa patients who were treated with adjuvant chemotherapy, female patients with non-zinc-binding mutations in *TP53* DNA-binding domain had the best 5-year survival compared to females with zinc-binding mutations or wildtype genotypes. The same impacts from *TP53* mutations on 5-year survival were not observed in men [30]. In addition, a polymorphism in *PLS3* gene was found to be a predictor of tumor recurrence time in female patients with stage-II/III CRCa receiving adjuvant chemotherapy [31]. In another study, it was reported that among inoperable non-small-cell LCa cases who received carboplatin and gemcitabine therapy, the lack of *ERCC1* gene expression conferred a survival advantage to male patients but not to females [29]. Additionally, women with advanced-staged non-small-cell LCa were more responsive to erlotinib, an *EGFR*-receptor inhibitor, compared to men which was partly attributed to higher mutations in *EGFR* in female patients [10,28]. Genetic factors may differentially impact chemotherapy-related toxicity as well. For instance, while women are more prone to fluorouracil (5-FU)-based chemotherapy toxicity [20], a splice site mutation in *DPYD* gene was strongly associated with sever 5-FU toxicity in men [32].

Despite the well-recognized sex differences in cancers, the underlying mechanisms have not been fully discovered and such disparities have not been consistently addressed in cancer research [5,22]. The evident contributions of genetic mechanisms to such sex disparities warrants further investigations into the sex-specific genetic architecture of LCa and CRCa, in particular due to their potential genetic heterogeneity. Exploring sex-specific genetic contributors to LCa and CRCa may provide more comprehensive insights into their underlying biological processes which in turn may help implementing more effective personalized and sex-specific medical interventions [5,10,22,33]. Searching genome-wide associations databases [34,35] shows that the genetic analysis of LCa’s and CRCa’s sex disparities has not received proper attention in previous GWAS. In this study, we performed sex-stratified genome-wide analyses of LCa and CRCa using phenotype and genotype data from three independent datasets to investigate potential sex disparities in the genetic predisposition to these common cancers.

## 2. Materials and Methods

### 2.1. Study Participants

**Figure 1:**
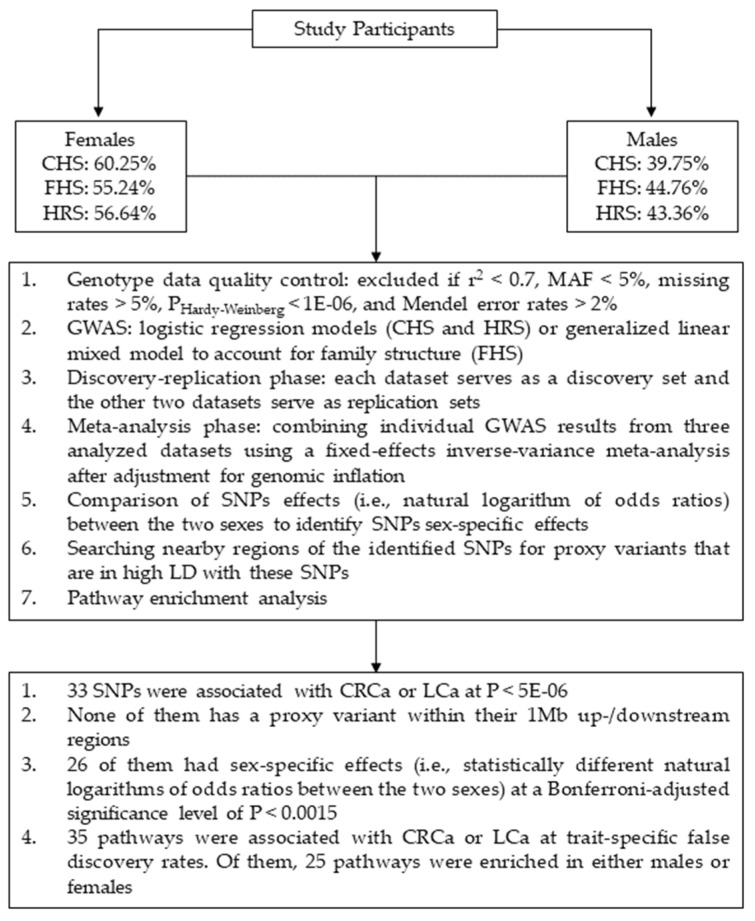
Schematic summary of the analysis steps and main findings of this study. Abbreviations: CHS = Cardiovascular Health Study; FHS = Framingham Heart Study; HRS = Health and Retirement Study; CRCa = colorectal cancer; LCa = lung cancer; SNP = single-nucleotide polymorphism; MAF = minor allele frequency; GWAS = genome-wide association study; LD = linkage disequilibrium.

Data from three independent studies were used including: Cardiovascular Health Study (CHS) [36], Framingham Heart Study (FHS) [37,38], and Health and Retirement Study (HRS) [39]. In each dataset, the genetic analyses were performed separately in females (i.e., CRCa-F and LCa-F) and males (i.e., CRCa-M and LCa-M). The cases comprised of 211 and 237 females as well as 186 and 220 males with CRCa and LCa, respectively. Also, 8382 and 8354 unaffected females and 6312 and 6278 unaffected males were included as controls in the CRCa-F, LCa-F, CRCa-M, and LCa-M analyses, respectively. The cases and controls were identified either by the study researchers (FHS) or by decoding medical diagnoses (CHS) or Medicare claims (HRS) using the International Classification of Disease codes, Ninth revision (ICD-9). Our genetic analyses were performed on subjects of Caucasian ancestry as there were not sufficient samples from other ethnicities. Table S1 (Supplementary File 1) provides summary demographic information for these three datasets. Figure 1 displays an overview of the analysis steps and main findings of our study.

### 2.2. Genotype Data and Quality Control (QC)

Our study made use of ~2 million genotyped and imputed SNPs. The imputation process has been detailed in [40]. Low-quality data were first filtered out including: (1) SNPs with imputation *r*^2^ < 0.7, (2) SNPs with minor allele frequencies (MAF) <5%, (3) SNPs/subjects with missing rates >5%, (4) SNPs deviated from Hardy–Weinberg equilibrium at *P* < 1.0 × 10^−6^, and (5) SNPs and subjects/families with Mendel error rates >2% in the case of FHS which is a family-based study. QC was performed using *PLINK* package [41]. This resulted in ~1.3–1.7 million SNPs in the datasets under consideration (Supplementary File 1: Table S2).

### 2.3. GWAS

#### 2.3.1. Genetic Models

Additive genetic models were fitted using *PLINK* package [41] to identify the association between SNPs and cancers of interest after adjustment for birth year, smoking history, and body mass index (BMI) of subjects, and the top 3–4 principal components of genotype data obtained by *GENESIS* R package [42]. To address the risk of inflation of type-I errors due to ignoring family structure [43], SNPs nominally (i.e., *P* < 0.05) associated with CRCa/LCa in FHS were reanalyzed by fitting generalized linear mixed models (using *lme4* R package [44]) which contained family IDs as a random-effects covariate in addition to the fixed-effects covariates stated above [40,45]. The results of GWAS of each cancer from the three datasets under consideration were then combined through an inverse-variance meta-analysis after adjustment for genomic inflation (i.e., λ values). Meta-analysis was performed using *GWAMA* package [46].

#### 2.3.2. Discovery and Replication Analyses 

We followed a commonly used discovery-replication strategy considering each of CHS, FHS, and HRS as a discovery set and the other two datasets as its counterpart replication sets. An association signal was considered replicated if a SNP had *P* < 5.0 × 10^−8^ (i.e., genome-wide significance) or 5.0 × 10^−8^ ≤ *P* < 5.0 × 10^−6^ (i.e., suggestive significance) [40] in GWAS of one dataset and *P* < 0.05 in another dataset, and had consist directions of associations in the discovery and replication sets. The SNPs that were not among the replicated set of SNPs but had significant P-values at genome-wide or suggestive significance levels in conducted meta-analyses constituted the meta-analysis set of significant SNPs.

#### 2.3.3. Novel Associations

CRCa/LCa-associated SNPs were considered as newly detected cancer variants if they were not associated with CRCa/LCa at *P* < 5.0 × 10^−6^ by previous GWAS available at databases such as GRASP [34] and NHGRI-EBI GWAS catalog [35]. *LDlink* web-tool [47] was then used to search possible proxy variants for the newly detected SNPs in the CEU population (i.e., Utah Residents with Northern and Western European Ancestry). A proxy variant was defined as a SNP that was located within ±1 Mb of a newly detected CRCa/LCa-associated SNP, was in LD with it (i.e., significant *𝒳^2^* in LD test) and was previously associated with the same cancer at *P* < 5.0 × 10^−6^.

#### 2.3.4. Sex-specific Associations 

SNPs disparately associated with CRCa/LCa in males and females were further analyzed by contrasting SNPs effects between males and females to determine if their effects were sex-specific [48]:

(1) $$\chi^{2}=\frac{{\left(b_{f}-b_{m}\right)}^{2}}{se_{f}^{2}+se_{m}^{2}}$$

where *χ*^2^ is the Wald’s Chi-square statistics*, b_f_* and *b_m_* are the SNP effects (i.e., the natural logarithm of odds ratios) in females and males, and *se_f_* and *se_m_* are their standard errors. 

### 2.4. Pathway Enrichment Analysis

Pathway enrichment analyses were performed by the *GSA-SNP2* package [49] using compound gene-based P-values, obtained according to the fastBAT method [50,51], to identify potential biological processes associated with the studied cancers in males and females. The canonical pathways from the Broad Institute gene set enrichment analysis (GSEA) [52] were considered as the reference pathways [53,54,55,56]. The significant pathways were determined at false discovery rates (FDR) [57] of 0.025 (CRCa-F and CRCa-M) and 0.05 (LCa-F and LCa-M) to keep the numbers of possible false-positive findings below one in each analyzed cancer.

## 3. Results

### 3.1. Fixed-Effects Covariates 

Smoking history, birth year, and BMI were included as fixed-effects covariates in our GWAS models to address their potential confounding effects on SNPs effects estimates, particularly due to their different distributions between males and females (Supplementary File 1: Table S1). Our meta-analyses revealed that smoking history and birth year were associated with CRCa and LCa in both males and females (*P* < 1.69 × 10^−2^) and BMI was associated with CRCa in both sexes (*P* < 7.74 ×10^−3^). However, their effects were not statistically different when their odds ratios were compared between males and females (Supplementary File 1: Table S3). 

### 3.2. GWAS 

Figures S1–S8 (Supplementary File 1) display the Manhattan and QQ plots from our GWAS. The λ values were smaller than 1.036 in these analyses (Supplementary File 1: Table S2), indicating the adequacy of population structure control [58]. Table 1 and Table S4 (Supplementary File 1) contain summary and detailed information regarding significant associations detected in our GWAS. We found that five SNPs (i.e., rs7593032, rs11000463, and rs11000467 in LCa-F; and rs9579517 and rs56357430 in CRCa-M) were associated with cancers of interest in a discovery dataset at suggestive significance level (i.e., 5.0 × 10^−8^ ≤ *P* < 5.0 × 10^−6^) and were replicated at *P* < 0.05 in a replication dataset with the same directions of effects. In addition, there were 28 SNPs which were associated with CRCa or LCa in conducted meta-analyses (P_META_ = 3.21 × 10^−7^ to 4.98 × 10^−6^; P_Q_ = 1.72× 10^−1^ to 9.75 × 10^−1^; and *i*^2^ values between 0 and 0.432). As seen in Table 1, there were several genes (i.e., *GLRX3* (CRCa-F), *PRKG1* (LCa-F), *MPHOSPH8* (CRCa-M), *LINC02039*, *MAP7*, and *GRIK1* (LCa-M)) to which multiple significant associations signals were mapped. SNPs mapped to each of these genes were in high LD (0.855 ≤ *r*^2^ ≤ 1 and D’=1) with each other in the CEU population [47] (Supplementary File 1: Table S5).

None of the 33 detected SNPs and their corresponding chromosomal regions had significant association signals in both sexes. Of these, 26 SNPs were sex-specific as their effect sizes (i.e., the natural logarithm of odds ratios) were statistically different between males and females at a Bonferroni-adjusted significance level of 0.0015 (i.e., 0.05/33) when compared by a Wald’s Chi-square test (Table 2).

### 3.3. Pathway Enrichment Analysis

Our analyses (Table 3) revealed that 11 and 13 pathways were significantly associated with LCa-F and LCa-M, respectively, at an FDR of 0.05. They were mainly involved in meiosis, chromosome maintenance and telomere/centromere organization, and DNA transcription. Of these, eight pathways were significant in both sexes, while three pathways in females and five pathways in males were specifically enriched in one sex. We also found that 19 pathways were associated with CRCa-M at an FDR of 0.025. They were mainly involved in immune system responses and signal transduction. No pathway was enriched in CRCa-F analyses at FDRs of 0.025 or 0.05, however, 2 pathways were significant at FDR of 0.2. These 2 pathways were among the 19 CRCa-M-associated pathways. The other 17 pathways detected in CRCa-M were male-specific. None of the detected pathways were associated with both CRCa and LCa.

## 4. Discussion

Almost all complex diseases (including many cancer types) have manifested sex disparities in epidemiological and clinical studies (e.g., in incidence/prevalence rates or disease severity) [21] which may be due to the hormonal effects, lifestyle risk factors, and genetic mechanisms, among others [5,10,21,22,40]. Investigating sex disparities in the genetic mechanisms underlying complex disorders may have translational impacts on medical interventions and has been stressed by the National Institutes of Health (NIH) [5,10,22,33].

In this study, we analyzed potential sex disparities in the genetic architectures of CRCa and LCa in three independent datasets which, to the best of our knowledge, were not used previously for the study of sex-specific genetic contributions to the cancer phenotypes of interest. Our GWAS revealed replicated association signals of five SNPs at P < 5.0 × 10^−6^ that were associated with CRCa in males or LCa in females. In addition, 28 other SNPs were significantly associated with CRCa or LCa at suggestive significance level in conducted meta-analyses (Table 1 and Table S4). None of the detected SNPs attained genome-wide significance in our study. This might be due to the insufficient sample sizes of this study or the heterogeneity of SNPs effects in the studied cohorts (i.e., CHS, FHS, and HRS). All these 33 SNPs were potentially novel markers for the studied cancers as their association signals were not reported by previous GWAS and, in addition, there were no proxy CRCa/LCa-associated SNPs within their ±1 Mb flanking regions. It should be noted that the significant associations detected in GWAS do not imply causality. Functional studies are needed to investigate whether the identified SNPs themselves or other variants in nearby chromosomal regions that are in high LD with these index SNPs contribute to the genetic architecture of the studied cancers. A literature review further delineated potential implications of these SNPs, their closest genes, and variants in nearby regions in CRCa and LCa. We found that the closest genes to these SNPs were not associated with the same cancer at P < 5.0 × 10^−6^ by previous GWAS [34,35], except for *CSMD1* gene (corresponding to rs13261356 detected in LCa-M) that was previously associated with LCa at genome-wide significance level [34,59]. Therefore, they can be considered as potentially novel genes for the studied cancers. However, nine of these genes (i.e., *KHDRBS3*, *PRKG1*, *ZRANB1*, *THRB*, *FSTL4*, *HTR1E*, *LINC01377*, *MIR4675*, and *GRIK1*) were previously implicated in cancers at other sites (i.e., other than those in our study) at P_GWAS_ < 5.0 ×10^−6^ [34,35], and 7 genes (i.e., *SNX31*, *KHDRBS3*, *GLRX3*, *THRB*, *MAP7*, *ATP8B1*, and *GRIK1*) were prognostically linked to other cancers at *P* < 0.001 (The Human Protein Atlas [60]: www.proteinatlas.org accessed on 2019–2020) (Table 1). In addition, 21 of 33 detected SNPs were located within nine chromosomal regions which were not previously associated with the same cancers at P_GWAS_ < 5.0 × 10^−6^ (i.e., *KHDRBS3*/8q24.23 and *GLRX3*/10q26.3 (CRCa-F); *PRKG1*/10q21.1 (LCa-F); *THRB*/3p24.2, *HTR1E*/6q14.3, and *MPHOSPH8*/13q12.11 (CRCa-M); *MAP7*/6q23.3, *ATP8B1*/18q21.31, and *GRIK1*/21q21.3 (LCa-M)).

Notably, 26 SNPs had sex-specific effects as they were significant only in males or females and their odds ratios were statistically different between the two sexes (Table 2). None of these SNPs were among or in LD with previously reported sex-linked SNPs [61,62]. The sex-specific SNPs associations with CRCa and LCa may advance the understanding of the underlying mechanisms of these common cancers in the two sexes by guiding functional studies in the detected chromosomal regions. Such sex-specific genetic factors may have translational implications in the era of personalized medicine by providing more efficient and cost-effective sex-specific medical interventions.

Our pathway enrichment analyses (Table 3) revealed that several pathways were significantly associated with CRCa and LCa (19 and 16 pathways, respectively). Most of the CRCa-associated pathways were related to the immune system functions. The intact/dysfunctional immune system responses were previously implicated in preventing/promoting tumorigenesis of CRCa [63,64]. The LCa-associated pathways were mostly involved in DNA replication/transcription and chromosome stability whose potential roles in LCa were previously highlighted [65,66,67]. Sex disparities were also noticed at the pathway level as most of the significant pathways (i.e., 17 pathways in CRCa and 8 pathways in LCa) were sex-specifically associated with the cancers of interest.

Limitations. Our analyses were generally underpowered for detecting association signals of SNPs with very small effect sizes and/or low MAFs (e.g., <0.05). Analyzing datasets with larger sample sizes would provide more statistical power and may replicate some of the detected disparate associations at genome-wide significance level and discover additional sex-specific associations. In addition, investigating potential sex disparities that may exist in the genetic architecture of different stages and/or histopathologic subtypes of CRCa and LCa may increase our knowledge about the genetic heterogeneity of these common cancers, although this requires sufficiently large sample sizes and availability of the staging and histopathologic data for the analyzed patients. 

## 5. Conclusions

Our genome-wide analyses revealed associations of 33 SNPs (mapped to 19 genes) with CRCa or LCa at suggestive significance levels which were significant in either males or females. None of these associations were reported by previous GWAS, and there were no proxy SNPs within ±1 Mb regions of the identified SNPs. Of these, 26 SNPs had sex-specific effects evidenced by significantly different effect sizes (i.e., the natural logarithm of odds ratios) between the two sexes. Our pathway enrichment analyses revealed 35 pathways, mainly involved in immune system functions, DNA replication/transcription, and chromosome stability, were associated with the studied cancers. Twenty-five of these pathways were significant in either males or females. The potential sex-specific contributions to the genetic architecture of CRCa and LCa identified in our study provided novel insights into the genetic heterogeneity of these common cancers, although they did not imply causality. Such sex-specific associations, if replicated in independent genome-wide studies and/or corroborated in functional studies, may have translational impacts on the medical interventions in CRCa and LCa.

## Figures and Tables

**Table 1:** Significant findings from genome-wide association analyses.

Chr	Gene	SNP	Pos	A1	P_HRS_	P_FHS_	P_CHS_	Effects	Freq	OR (se)	P_META_	P_Q_	*i* ^2^	N	1Mb?	Proxy?	Region?	Gene?	Prognostic?
**CRCa-F**																			
8q22.3	*SNX31*	rs1078186	100613251	C	4.19 × 10^−4^	9.48 × 10^−4^	2.21 × 10^−1^	---	0.574	0.615 (0.056)	1.60 × 10^−6^	4.49 × 10^−1^	0	8590	N	N	S	N	O
8q24.23	*KHDRBS3*	rs118174020	135843271	C	NA	3.92 × 10^−4^	8.73 × 10^−4^	?--	0.949	0.357 (0.062)	1.41 × 10^−6^	8.49 × 10^−1^	0	4752	N	N	N	O	O
10q26.3	*GLRX3*	rs3118492	130435775	A	2.74 × 10^−3^	8.05 × 10^−3^	1.69 × 10^−2^	+++	0.229	1.672 (0.168)	4.15 × 10^−6^	9.71 × 10^−1^	0	8544	N	N	N	N	O
10q26.3	*GLRX3*	rs372813	130442933	A	2.89 × 10^−3^	6.08 × 10^−3^	1.68 × 10^−2^	+++	0.233	1.661 (0.163)	3.25 × 10^−6^	9.75 × 10^−1^	0	8592	N	N	N	N	O
10q26.3	*GLRX3*	rs359063	130452326	T	4.64 × 10^−3^	3.89 × 10^−3^	1.39 × 10^−2^	+++	0.230	1.669 (0.164)	2.86 × 10^−6^	9.14 × 10^−1^	0	8545	N	N	N	N	O
10q26.3	*GLRX3*	rs307385	130452402	T	3.61 × 10^−3^	3.50 × 10^−3^	1.39 × 10^−2^	+++	0.230	1.684 (0.167)	2.16 × 10^−6^	9.23 × 10^−1^	0	8543	N	N	N	N	O
**LCa-F**																			
2p21	*LOC102723824*	rs7593032 *	42122050	T	4.63 × 10^−6^	3.32 × 10^−1^	3.68 × 10^−2^	---	0.713	0.631 (0.057)	3.66 × 10^−6^	9.14 × 10^−2^	0.582	8577	N	N	S	N	N
5q13.2	*LINC02056*	rs42775	72584687	T	2.43 × 10^−4^	1.97 × 10^−2^	8.54 × 10^−2^	+++	0.325	1.561 (0.138)	4.98 × 10^−6^	6.74 × 10^−1^	0	8516	S	N	S	N	N
10q21.1	*PRKG1*	rs11000463 *	52014359	T	4.60 × 10^−6^	3.77 × 10^−2^	7.65 × 10^−1^	--+	0.903	0.558 (0.067)	1.75 × 10^−5^	3.31 × 10^−2^	0.707	8591	N	N	N	O	N
10q21.1	*PRKG1*	rs11000467 *	52015312	G	4.67 × 10^−6^	2.87 × 10^−2^	7.65 × 10^−1^	--+	0.905	0.548 (0.066)	1.37 × 10^−5^	3.41 × 10^−2^	0.704	8570	N	N	N	O	N
10q26.13	*ZRANB1*	rs76972397	124961380	T	1.70 × 10^−4^	NA	1.43 × 10^−3^	-?-	0.930	0.432 (0.063)	9.62 × 10^−7^	6.72 × 10^−1^	0	5770	N	N	G	O	N
18q11.2	*HRH4*	rs482962	24532843	A	2.41 × 10^−2^	2.04 × 10^−3^	4.52 × 10^−3^	+++	0.262	1.607 (0.151)	4.90 × 10^−6^	4.75 × 10^−1^	0	8585	N	N	S	N	N
**CRCa-M**																			
3p24.2	*THRB*	rs57751578	24486090	T	5.16 × 10^−4^	5.80 × 10^−2^	4.39 × 10^−3^	---	0.911	0.483 (0.064)	1.89 × 10^−6^	6.98 × 10^−1^	0	6491	N	N	N	O	O
5q31.1	*FSTL4*	rs4631227	133152748	A	1.81 × 10^−4^	5.77 × 10^−2^	1.49 × 10^−2^	+++	0.293	1.663 (0.163)	3.20 × 10^−6^	4.76 × 10^−1^	0	6481	N	N	G	O	N
6q14.3	*HTR1E*	rs72907251	86515650	C	7.49 × 10^−5^	1.55 × 10^−3^	4.64 × 10^−1^	---	0.937	0.446 (0.063)	1.06 × 10^−6^	3.73 × 10^−1^	0	6496	N	N	N	O	N
13q12.11	*MPHOSPH8*	rs9579517 *	19603453	C	2.43 × 10^−6^	8.91 × 10^−1^	2.62 × 10^−2^	---	0.900	0.519 (0.066)	8.88 × 10^−6^	3.31 × 10^−2^	0.707	6490	N	N	N	N	N
13q12.11	*MPHOSPH8*	rs56357430 *	19632421	C	2.43 × 10^−6^	9.20 × 10^−1^	3.69 × 10^−2^	-+-	0.900	0.528 (0.068)	1.77 × 10^−5^	2.25 × 10^−2^	0.736	6498	N	N	N	N	N
**LCa-M**																			
5p15.33	*LINC01377*	rs12657742	3123661	A	5.98 × 10^−3^	2.66 × 10^−3^	2.27 × 10^−2^	---	0.864	0.555 (0.062)	3.67 × 10^−6^	9.63 × 10^−1^	0	6490	N	N	G	O	N
5q23.2	*LINC02039*	rs77914729	126003571	C	6.07 × 10^−3^	1.33 × 10^−3^	2.67 × 10^−2^	---	0.929	0.475 (0.065)	2.67 × 10^−6^	8.09 × 10^−1^	0	6495	N	N	S	N	N
5q23.2	*LINC02039*	rs75874914	126003878	C	6.25 × 10^−3^	1.64 × 10^−3^	2.66 × 10^−2^	---	0.929	0.478 (0.065)	3.24 × 10^−6^	8.29 × 10^−1^	0	6496	N	N	S	N	N
5q23.2	*LINC02039*	rs11954381	126006435	G	5.32 × 10^−3^	1.73 × 10^−3^	2.66 × 10^−2^	---	0.929	0.477 (0.065)	2.81 × 10^−6^	8.63 × 10^−1^	0	6491	N	N	S	N	N
6q23.3	*MAP7*	rs9399183	136496350	A	1.03 × 10^−5^	7.56 × 10^−2^	1.33 × 10^−1^	---	0.833	0.585 (0.060)	3.33 × 10^−6^	2.19 × 10^−1^	0.341	6494	N	N	N	N	O
6q23.3	*MAP7*	rs3799451	136508794	A	4.91 × 10^−6^	5.78 × 10^−2^	1.61 × 10^−1^	---	0.852	0.565 (0.060)	1.62 × 10^−6^	2.01 × 10^−1^	0.377	6495	N	N	N	N	O
6q23.3	*MAP7*	rs3799453	136518050	G	4.70 × 10^−6^	5.78 × 10^−2^	2.42 × 10^−1^	---	0.853	0.570 (0.061)	2.46 × 10^−6^	1.72 × 10^−1^	0.432	6495	N	N	N	N	O
6q23.3	*MAP7*	rs3799454	136518078	T	6.90 × 10^−6^	6.85 × 10^−2^	2.16 × 10^−1^	---	0.835	0.586 (0.060)	3.58 × 10^−6^	1.76 × 10^−1^	0.425	6498	N	N	N	N	O
6q23.3	*MAP7*	rs3799462	136524611	G	1.10 × 10^−5^	5.78 × 10^−2^	2.42 × 10^−1^	---	0.853	0.577 (0.062)	4.49 × 10^−6^	2.12 × 10^−1^	0.356	6498	N	N	N	N	O
8p23.2	*CSMD1*	rs13261356	4605119	C	1.04 × 10^−3^	8.05 × 10^−4^	NA	--?	0.900	0.482 (0.065)	3.23 × 10^−6^	7.69 × 10^−1^	0	5209	G	N	G	SO	N
10p12.31	*MIR4675*	rs11012129	20545511	T	2.15 × 10^−3^	1.68 × 10^−5^	NA	--?	0.945	0.375 (0.060)	3.21 × 10^−7^	2.14 × 10^−1^	0.352	5207	N	N	S	O	N
18q21.31	*ATP8B1*	rs2437037	57795758	A	2.39 × 10^−4^	NA	4.50 × 10^−3^	-?-	0.909	0.449 (0.066)	3.95 × 10^−6^	8.71 × 10^−1^	0	4216	N	N	N	N	O
21q21.3	*GRIK1*	rs363433	29588811	C	8.08 × 10^−4^	1.03 × 10^−2^	1.20 × 10^−2^	---	0.863	0.539 (0.061)	1.14 × 10^−6^	9.30 × 10^−1^	0	6490	N	N	N	O	O
21q21.3	*GRIK1*	rs363432	29591270	G	3.23 × 10^−3^	1.03 × 10^−2^	1.12 × 10^−2^	---	0.863	0.554 (0.063)	4.10 × 10^−6^	9.03 × 10^−1^	0	6495	N	N	N	O	O
21q21.3	*GRIK1*	rs2832405	29591425	T	5.58 × 10^−4^	1.24 × 10^−2^	1.98 × 10^−2^	---	0.854	0.548 (0.061)	1.50 × 10^−6^	9.16 × 10^−1^	0	6497	N	N	N	O	O
21q21.3	*GRIK1*	rs363472	29595793	G	1.77 × 10^−3^	1.29 × 10^−2^	1.78 × 10^−2^	---	0.856	0.562 (0.062)	4.23 × 10^−6^	9.26 × 10^−1^	0	6490	N	N	N	O	O

Abbreviations: SNP = single-nucleotide polymorphism; CRCa-F = colorectal cancer in females; LCa-F = lung cancer in females; CRCa-M = colorectal cancer in males; LCa-M = lung cancer in males; Chr = chromosomal region (i.e., cytogenetic band); Gene = the gene within which the SNP is located or else the closest gene to the SNP; Pos = SNP position based on Human Genome version 38 (hg38); A1 = effect allele; P_HRS_, P_FHS_, and P_CHS_ = P-values in HRS (Health and Retirement Study), FHS (Framingham Heart Study), and CHS (Cardiovascular Health Study) datasets, respectively; Effects = directions of SNP’s effects in the aforementioned datasets (Positive, Negative, Missing); Freq = frequency of effect allele in conducted meta-analysis; OR (se) = odds ratio and its standard error in conducted meta-analysis; P_META_ = *P*-value in conducted meta-analysis; P_Q_ = P-value of Q-statistics (Cochran’s heterogeneity test); *i*^2^ = i-squared inconsistency metric; N = Number of subjects with non-missing genotype data in conducted meta-analysis; 1Mb? = whether any polymorphism associated with the same cancer was previously discovered within the ±1 Mb of the SNP detected here (G: polymorphism with *P* < 5.0 ×10^−8^, and S: polymorphism with 5.0 × 10^−8^ ≤ *P* < 5.0 × 10^−6^, N: none); Proxy? = whether any of the 1Mb?-SNPs is in linkage disequilibrium (LD) with the SNP detected here and its P-value is less than the one detected in this study (Y: yes, N: no); Region? = whether any SNP within the chromosomal region corresponding to the SNP detected here was previously associated with the same cancer at *P* < 5.0 × 10^−6^ (Y: yes, N: no); Gene? = whether any SNP mapped to the gene corresponding to the SNP detected here was previously associated with cancers at *P* < 5.0 × 10^−6^ (S: same cancer, O: other cancers, N: none); Prognostic? = whether the closest gene to the SNP detected here was previously reported as a cancer prognostic factor at *P* < 0.001 (S: same cancer, O: other cancers, N: none); * = Replicated SNPs.

**Table 2:** Wald’s Chi-square test to compare odds ratios of detected cancer-associated SNPs between males and females.

Chr	Gene	SNP	Pos	A1	Males				Females				Comparison	
					Freq	OR	se	*P*-Value	Freq	OR	se	*P*-Value	Chi-Square	*P*-Value
**CRCa-F**														
8q22.3	*SNX31*	rs1078186	100613251	C	0.568	0.989	0.097	9.19 × 10^−1^	0.574	0.615	0.056	1.60 × 10^−6^	18.033	2.17 × 10^−5^
8q24.23	*KHDRBS3*	rs118174020 ^+^	135843271	C	NA	NA	NA	NA	0.949	0.357	0.062	1.41 × 10^−6^	NA	NA
10q26.3	*GLRX3*	rs3118492 ^+^	130435775	A	0.235	1.032	0.119	8.07 × 10^−1^	0.229	1.672	0.168	4.15 × 10^−6^	5.507	1.89 × 10^−2^
10q26.3	*GLRX3*	rs372813 ^+^	130442933	A	0.238	1.037	0.118	7.79 × 10^−1^	0.233	1.661	0.163	3.25 × 10^−6^	5.497	1.90 × 10^−2^
10q26.3	*GLRX3*	rs359063 ^+^	130452326	T	0.235	1.061	0.121	6.43 × 10^−1^	0.230	1.669	0.164	2.86 × 10^−6^	4.935	2.63 × 10^−2^
10q26.3	*GLRX3*	rs307385 ^+^	130452402	T	0.234	1.066	0.122	6.21 × 10^−1^	0.230	1.684	0.167	2.16 × 10^−6^	4.923	2.65 × 10^−2^
**LCa-F**														
2p21	*LOC102723824*	rs7593032	42122050	T	0.711	1.206	0.122	9.73 × 10^−2^	0.713	0.631	0.057	3.66 × 10^−6^	23.096	1.54 × 10^−6^
5q13.2	*LOC102477328*	rs42775 ^+^	72584687	T	0.326	0.958	0.093	6.92 × 10^−1^	0.325	1.561	0.138	4.98 × 10^−6^	8.542	3.47 × 10^−3^
10q21.1	*PRKG1*	rs11000463	52014359	T	0.903	1.266	0.199	2.09 × 10^−1^	0.903	0.558	0.067	1.75 × 10^−5^	15.245	9.44 × 10^−5^
10q21.1	*PRKG1*	rs11000467	52015312	G	0.904	1.252	0.201	2.43 × 10^−1^	0.905	0.548	0.066	1.37 × 10^−5^	15.283	9.25 × 10^−5^
10q26.13	*ZRANB1*	rs76972397	124961380	T	0.935	0.945	0.193	8.27 × 10^−1^	0.930	0.432	0.063	9.62 × 10^−7^	14.928	1.12 × 10^−4^
18q11.2	*HRH4*	rs482962^+^	24532843	A	0.252	1.132	0.116	2.76 × 10^−1^	0.262	1.607	0.151	4.90 × 10^−6^	3.390	6.56 × 10^−2^
**CRCa-M**														
3p24.2	*THRB*	rs57751578	24486090	T	0.911	0.483	0.064	1.89 × 10^−6^	0.909	1.131	0.175	5.03 × 10^−1^	20.847	4.97 × 10^−6^
5q31.1	*FSTL4*	rs4631227	133152748	A	0.293	1.663	0.163	3.20 × 10^−6^	0.293	0.892	0.090	3.09 × 10^−1^	11.149	8.41 × 10^−4^
6q14.3	*HTR1E*	rs72907251	86515650	C	0.937	0.446	0.063	1.06 × 10^−6^	0.935	0.774	0.122	1.74 × 10^−1^	16.133	5.91 × 10^−5^
13q12.11	*MPHOSPH8*	rs9579517	19603453	C	0.900	0.519	0.066	8.88 × 10^−6^	0.901	0.919	0.13	6.11 × 10^−1^	15.390	8.74 × 10^−5^
13q12.11	*MPHOSPH8*	rs56357430	19632421	C	0.900	0.528	0.068	1.77 × 10^−5^	0.901	0.915	0.128	5.89 × 10^−1^	14.294	1.56 × 10^−4^
**LCa-M**														
5p15.33	*LOC102467074*	rs12657742	3123661	A	0.864	0.555	0.062	3.67 × 10^−6^	0.868	0.975	0.118	8.56 × 10^−1^	17.809	2.44 × 10^−5^
5q23.2	*LOC102546228*	rs77914729	126003571	C	0.929	0.475	0.065	2.67 × 10^−6^	0.932	1.047	0.173	8.16 × 10^−1^	18.396	1.79 × 10^−5^
5q23.2	*LOC102546228*	rs75874914	126003878	C	0.929	0.478	0.065	3.24 × 10^−6^	0.932	1.095	0.182	6.51 × 10^−1^	18.307	1.88 × 10^−5^
5q23.2	*LOC102546228*	rs11954381	126006435	G	0.929	0.477	0.065	2.81 × 10^−6^	0.932	1.133	0.191	5.41 × 10^−1^	18.408	1.78 × 10^−5^
6q23.3	*MAP7*	rs9399183	136496350	A	0.833	0.585	0.060	3.33 × 10^−6^	0.838	0.863	0.097	2.43 × 10^−1^	11.669	6.36 × 10^−4^
6q23.3	*MAP7*	rs3799451	136508794	A	0.852	0.565	0.060	1.62 × 10^−6^	0.854	0.833	0.095	1.57 × 10^−1^	11.877	5.68 × 10^−4^
6q23.3	*MAP7*	rs3799453	136518050	G	0.853	0.570	0.061	2.46 × 10^−6^	0.854	0.832	0.095	1.54 × 10^−1^	11.287	7.81 × 10^−4^
6q23.3	*MAP7*	rs3799454	136518078	T	0.835	0.586	0.060	3.58 × 10^−6^	0.840	0.852	0.095	2.03 × 10^−1^	11.010	9.06 × 10^−4^
6q23.3	*MAP7*	rs3799462	136524611	G	0.853	0.577	0.062	4.49 × 10^−6^	0.855	0.829	0.095	1.47 × 10^−1^	10.291	1.34 × 10^−3^
8p23.2	*CSMD1*	rs13261356	4605119	C	0.900	0.482	0.065	3.23 × 10^−6^	0.903	0.852	0.128	3.68 × 10^−1^	15.839	6.90 × 10^−5^
10p12.31	*MIR4675*	rs11012129	20545511	T	0.945	0.375	0.060	3.21 × 10^−7^	0.945	1.465	0.288	1.23 × 10^−1^	21.542	3.46 × 10^−6^
18q21.31	*ATP8B1*	rs2437037	57795758	A	0.909	0.449	0.066	3.95 × 10^−6^	0.910	1.075	0.185	7.30 × 10^−1^	19.804	8.58 × 10^−6^
21q21.3	*GRIK1*	rs363433	29588811	C	0.863	0.539	0.061	1.14 × 10^−6^	0.861	0.882	0.102	3.40 × 10^−1^	17.206	3.35 × 10^−5^
21q21.3	*GRIK1*	rs363432	29591270	G	0.863	0.554	0.063	4.10 × 10^−6^	0.861	0.879	0.102	3.26 × 10^−1^	14.882	1.14 × 10^−4^
21q21.3	*GRIK1*	rs2832405	29591425	T	0.854	0.548	0.061	1.50 × 10^−6^	0.853	0.924	0.106	5.45 × 10^−1^	18.197	1.99 × 10^−5^
21q21.3	*GRIK1*	rs363472	29595793	G	0.856	0.562	0.062	4.23 × 10^−6^	0.855	0.895	0.102	3.89 × 10^−1^	15.071	1.04 × 10^−4^

Please see the description provided below Table 1. + denotes the SNP did not have sex-specific effects (i.e., *P*-value ≥ 0.0015 in Chi-square test comparing SNP effects in males and females).

**Table 3:** Significant findings from pathway enrichment analyses.

Pathway	Pathway Source	GSEA ID	Size	Males				Females			
				Count	*z*-Score	*p*-Value	*q*-Value	Count	*z*-Score	*p*-Value	*q*-Value
**CRCa**											
*Regulation of IFNA signaling*	REACTOME	M982	24	24	8.33	0	0	24	3.64	1.36 × 10^−4^	1.72 × 10^−1^
*Antigen processing and presentation*	KEGG	M16004	89	84	7.69	6.83 × 10^−15^	4.32 × 10^−12^	NS	NS	NS	NS
*Natural killer cell mediated cytotoxicity*	KEGG	M5669	137	135	6.71	9.61 × 10^−12^	4.05 × 10^−9^	NS	NS	NS	NS
*Regulation of autophagy*	KEGG	M6382	35	35	5.67	7.09 × 10^−9^	2.24 × 10^−6^	NS	NS	NS	NS
*TRAF6 mediated IRF7 activation*	REACTOME	M936	30	30	5.63	9.24 × 10^−9^	2.34 × 10^−6^	NS	NS	NS	NS
*Autoimmune thyroid disease*	KEGG	M13103	53	50	5.62	9.64 × 10^−9^	2.34 × 10^−6^	NS	NS	NS	NS
*CD8/TCR downstream signaling*	PID	M272	65	65	4.97	3.27 × 10^−7^	5.91 × 10^−5^	NS	NS	NS	NS
*Interferon alpha/beta signaling*	REACTOME	M973	64	62	4.78	8.82 × 10^−7^	1.40 × 10^−4^	NS	NS	NS	NS
*Toll-like receptor signaling pathway*	KEGG	M3261	102	100	4.54	2.76 × 10^−6^	3.88 × 10^−4^	100	3.51	2.26 × 10^−4^	1.72 × 10^−1^
*RIG-I-like receptor signaling pathway*	KEGG	M15913	71	69	4.29	8.86 × 10^−6^	1.12 × 10^−3^	NS	NS	NS	NS
*Translocation of ZAP-70 to immunological synapse*	REACTOME	M722	14	13	4.15	1.66 × 10^−5^	1.91 × 10^−3^	NS	NS	NS	NS
*JAK-STAT signaling pathway*	KEGG	M17411	155	146	4.07	2.32 × 10^−5^	2.44 × 10^−3^	NS	NS	NS	NS
*Immunoregulatory interactions between a lymphoid and a non-lymphoid cell*	REACTOME	M8240	70	64	3.91	4.68 × 10^−5^	4.56 × 10^−3^	NS	NS	NS	NS
*Generation of second messenger molecules*	REACTOME	M16523	27	26	3.84	6.05 × 10^−5^	5.46 × 10^−3^	NS	NS	NS	NS
*Cytosolic DNA-sensing pathway*	KEGG	M11844	56	55	3.69	1.10 × 10^−4^	9.28 × 10^−3^	NS	NS	NS	NS
*Phosphorylation of CD3 and TCR zeta chains*	REACTOME	M12494	16	15	3.68	1.18 × 10^−4^	9.33 × 10^−3^	NS	NS	NS	NS
*Interferon signaling*	REACTOME	M983	159	152	3.67	1.21 × 10^−4^	9.33 × 10^−3^	NS	NS	NS	NS
*P2Y receptors*	REACTOME	M10960	12	12	3.59	1.66 × 10^−4^	1.17 × 10^−2^	NS	NS	NS	NS
*PD-1 signaling*	REACTOME	M18810	18	17	3.41	3.19 × 10^−4^	2.13 × 10^−2^	NS	NS	NS	NS
**LCa**											
*RNA polymerase I promoter opening*	REACTOME	M884	62	59	5.52	1.67 × 10^−8^	2.11 × 10^−5^	59	5.78	3.83 × 10^−9^	4.84 × 10^−6^
*Meiotic recombination*	REACTOME	M1011	86	82	4.83	6.77 × 10^−7^	4.28 × 10^−4^	82	4.19	1.38 × 10^−5^	2.49 × 10^−3^
*Amyloid fiber formation*	REACTOME	M1076	83	79	4.68	1.42 × 10^−6^	4.97 × 10^−4^	79	4.63	1.80 × 10^−6^	5.69 × 10^−4^
*RNA polymerase I transcription*	REACTOME	M728	89	83	4.72	1.18 × 10^−6^	4.97 × 10^−4^	83	4.25	1.08 × 10^−5^	2.28 × 10^−3^
*Interleukin-7 signaling*	REACTOME	M542	11	11	4.46	4.04 × 10^−6^	1.02 × 10^−3^	NS	NS	NS	NS
*Meiosis*	REACTOME	M529	116	110	4.26	1.02 × 10^−5^	2.16 × 10^−3^	110	3.47	2.61 × 10^−4^	3.31 × 10^−2^
*Packaging of telomere ends*	REACTOME	M17695	48	48	4.19	1.42 × 10^−5^	2.57 × 10^−3^	48	5.47	2.20 × 10^−8^	1.39 × 10^−5^
*RNA polymerase I, RNA polymerase III, and mitochondrial transcription*	REACTOME	M858	122	116	3.99	3.36 × 10^−5^	5.31 × 10^−3^	NS	NS	NS	NS
*Meiotic synapsis*	REACTOME	M1061	73	71	3.88	5.20 × 10^−5^	7.32 × 10^−3^	71	4.31	8.14 × 10^−6^	2.06 × 10^−3^
*A6B1 and A6B4 integrin signaling*	PID	M239	46	46	3.83	6.29 × 10^−5^	7.95 × 10^−3^	NS	NS	NS	NS
*Interleukin-7 signaling*	BIOCARTA	M1296	17	17	3.62	1.49 × 10^−4^	1.71 × 10^−2^	NS	NS	NS	NS
*Interleukin-2 family signaling*	REACTOME	M1012	41	39	3.58	1.70 × 10^−4^	1.79 × 10^−2^	NS	NS	NS	NS
*Deposition of new CENPA-containing nucleosomes at the centromere*	REACTOME	M871	64	61	3.44	2.88 × 10^−4^	2.81 × 10^−2^	61	3.79	7.53 × 10^−5^	1.12 × 10^−2^
*Telomere maintenance*	REACTOME	M4052	75	NS	NS	NS	NS	75	5.04	2.37 × 10^−7^	1.00 × 10^−4^
*Systemic lupus erythematosus*	KEGG	M4741	140	NS	NS	NS	NS	133	3.81	7.09 × 10^−5^	1.12 × 10^−2^
*Chromosome maintenance*	REACTOME	M868	122	NS	NS	NS	NS	117	3.4	3.42 × 10^−4^	3.94 × 10^−2^

Abbreviations: CRCa = colorectal cancer; LCa = lung cancer; GSEA = Gene Set Enrichment Analysis Platform; KEGG = Kyoto Encyclopedia of Genes and Genomes [53]; BIOCARTA = BIOCARTA pathways [54]; PID = Pathway Interaction Database [55]; REACTOME = REACTOME pathway knowledgebase [56]; Size = number of genes in the pathway; Count = number of enriched genes in the pathway; NS = non-significant.

## Data Availability

The CHS and FHS datasets as well as the HRS genetic data were obtained from dbGaP (https://www.ncbi.nlm.nih.gov/gap, accession numbers: phs000287.v5.p1 (CHS), phs000007.v28.p10 (FHS), and phs000428.v1.p1 (HRS)). Phenotypic HRS data were obtained from the University of Michigan and the Centers for Medicare and Medical Services (CMS) in 2015–2016 and downloaded from the HRS public website (http://hrsonline.isr.umich.edu/index.php?p=data).

## Supplementary Materials

The following are available online at https://www.mdpi.com/article/10.3390/genes12050686/s1, Supplementary File 1: Supporting Acknowledgment, Table S1. Demographic information about the analyzed datasets; Table S2. Numbers (and percentages) of analyzed SNPs along with the genomic inflation factors (λ) resulted from our genome-wide association analyses; Table S3. The odds ratios of fixed-effects covariates in females and males from conducted meta-analyses; Table S4. Cancer-associated SNPs from genome-wide association analyses; Table S5. Linkage disequilibrium measures among SNPs with significant association signals that were mapped to the same gene; Figure S1. Manhattan plot of the genome-wide association analyses of colorectal cancer in females (CRCa-F); Figure S2. QQ plot of the genome-wide association analyses of colorectal cancer in females (CRCa-F); Figure S3. Manhattan plot of the genome-wide association analyses of lung cancer in females (LCa-F); Figure S4. QQ plot of the genome-wide association analyses of lung cancer in females (LCa-F); Figure S5. Manhattan plot of the genome-wide association analyses of colorectal cancer in males (CRCa-M); Figure S6. QQ plot of the genome-wide association analyses of colorectal cancer in males (CRCa-M); Figure S7. Manhattan plot of the genome-wide association analyses of lung cancer in males (LCa-M); Figure S8. QQ plot of the genome-wide association analyses of lung cancer in males (LCa-M).

## References

[1] Cook M.B., Dawsey S.M., Freedman N.D., Inskip P.D., Wichner S.M., Quraishi S.M., Devesa S.S., McGlynn K.A. (2009). Sex Disparities in Cancer Incidence by Period and Age. Cancer Epidemiol. Biomark. Prev..

[2] Cook M.B., McGlynn K.A., Devesa S.S., Freedman N.D., Anderson W.F. (2011). Sex Disparities in Cancer Mortality and Survival. Cancer Epidemiol. Biomark. Prev..

[3] Edgren G., Liang L., Adami H.-O., Chang E.T. (2012). Enigmatic Sex Disparities in Cancer Incidence. Eur. J. Epidemiol..

[4] Li C.H., Haider S., Shiah Y.-J., Thai K., Boutros P.C. (2018). Sex Differences in Cancer Driver Genes and Biomarkers. Cancer Res..

[5] Rubin J.B., Lagas J.S., Broestl L., Sponagel J., Rockwell N., Rhee G., Rosen S.F., Chen S., Klein R.S., Imoukhuede P. (2020). Sex Differences in Cancer Mechanisms. Biol. Sex Differ..

[6] Bray F., Ferlay J., Soerjomataram I., Siegel R.L., Torre L.A., Jemal A. (2018). Global Cancer Statistics 2018: GLOBOCAN Estimates of Incidence and Mortality Worldwide for 36 Cancers in 185 Countries. CA A Cancer J. Clin..

[7] Ferguson M.K., Skosey C., Hoffman P.C., Golomb H.M. (1990). Sex-Associated Differences in Presentation and Survival in Patients with Lung Cancer. J. Clin. Oncol.

[8] Singh S., Parulekar W., Murray N., Feld R., Evans W.K., Tu D., Shepherd F.A. (2005). Influence of Sex on Toxicity and Treatment Outcome in Small-Cell Lung Cancer. J. Clin. Oncol..

[9] Yang Y., Wang G., He J., Ren S., Wu F., Zhang J., Wang F. (2017). Gender Differences in Colorectal Cancer Survival: A Meta-Analysis. Int. J. Cancer.

[10] Pal S.K., Hurria A. (2010). Impact of Age, Sex, and Comorbidity on Cancer Therapy and Disease Progression. J. Clin. Oncol..

[11] Kiyohara C., Ohno Y. (2010). Sex Differences in Lung Cancer Susceptibility: A Review. Gend. Med..

[12] Kim S.-E., Paik H.Y., Yoon H., Lee J.E., Kim N., Sung M.-K. (2015). Sex- and Gender-Specific Disparities in Colorectal Cancer Risk. World J. Gastroenterol..

[13] Benedix F., Kube R., Meyer F., Schmidt U., Gastinger I., Lippert H. (2010). Colon/Rectum Carcinomas (Primary Tumor) Study Group Comparison of 17,641 Patients with Right- and Left-Sided Colon Cancer: Differences in Epidemiology, Perioperative Course, Histology, and Survival. Dis. Colon Rectum.

[14] Hansen I.O., Jess P. (2012). Possible Better Long-Term Survival in Left versus Right-Sided Colon Cancer—A Systematic Review. Dan Med. J..

[15] Cheng T.-Y.D., Cramb S.M., Baade P.D., Youlden D.R., Nwogu C., Reid M.E. (2016). The International Epidemiology of Lung Cancer: Latest Trends, Disparities, and Tumor Characteristics. J. Thorac. Oncol..

[16] Wilkinson I.B., Raine T., Wiles K., Goodhart A., Hall C., O’Neill H., Sinharay R., Furmedge D. (2019). Oxford Handbook of Clinical Medicine.

[17] Farach-Carson M.C., Lin S.-H., Nalty T., Satcher R.L. (2017). Sex Differences and Bone Metastases of Breast, Lung, and Prostate Cancers: Do Bone Homing Cancers Favor Feminized Bone Marrow?. Front. Oncol..

[18] Sun T., Warrington N.M., Rubin J.B. (2012). Why Does Jack, and Not Jill, Break His Crown? Sex Disparity in Brain Tumors. Biol. Sex Differ..

[19] Koo J.H., Jalaludin B., Wong S.K.C., Kneebone A., Connor S.J., Leong R.W.L. (2008). Improved Survival in Young Women with Colorectal Cancer. Off. J. Am. Coll. Gastroenterol..

[20] Sloan J.A., Goldberg R.M., Sargent D.J., Vargas-Chanes D., Nair S., Cha S.S., Novotny P.J., Poon M.A., O’Connell M.J., Loprinzi C.L. (2002). Women Experience Greater Toxicity with Fluorouracil-Based Chemotherapy for Colorectal Cancer. J. Clin. Oncol..

[21] Ober C., Loisel D.A., Gilad Y. (2008). Sex-Specific Genetic Architecture of Human Disease. Nat. Rev. Genet..

[22] Dorak M.T., Karpuzoglu E. (2012). Gender Differences in Cancer Susceptibility: An Inadequately Addressed Issue. Front. Genet..

[23] Galvan A., Ioannidis J.P.A., Dragani T.A. (2010). Beyond Genome-Wide Association Studies: Genetic Heterogeneity and Individual Predisposition to Cancer. Trends Genet..

[24] Vogelstein B., Papadopoulos N., Velculescu V.E., Zhou S., Diaz L.A., Kinzler K.W. (2013). Cancer Genome Landscapes. Science.

[25] Ulivi P., Scarpi E., Chiadini E., Marisi G., Valgiusti M., Capelli L., Casadei Gardini A., Monti M., Ruscelli S., Frassineti G.L. (2017). Right- vs. Left-Sided Metastatic Colorectal Cancer: Differences in Tumor Biology and Bevacizumab Efficacy. Int. J. Mol. Sci..

[26] Gaj P., Maryan N., Hennig E.E., Ledwon J.K., Paziewska A., Majewska A., Karczmarski J., Nesteruk M., Wolski J., Antoniewicz A.A. (2012). Pooled Sample-Based GWAS: A Cost-Effective Alternative for Identifying Colorectal and Prostate Cancer Risk Variants in the Polish Population. PLoS ONE.

[27] Timofeeva M.N., Hung R.J., Rafnar T., Christiani D.C., Field J.K., Bickeböller H., Risch A., McKay J.D., Wang Y., Dai J. (2012). Influence of Common Genetic Variation on Lung Cancer Risk: Meta-Analysis of 14 900 Cases and 29 485 Controls. Hum. Mol. Genet..

[28] Shepherd F.A., Rodrigues Pereira J., Ciuleanu T., Tan E.H., Hirsh V., Thongprasert S., Campos D., Maoleekoonpiroj S., Smylie M., Martins R. (2005). Erlotinib in Previously Treated Non-Small-Cell Lung Cancer. N. Engl. J. Med..

[29] Holm B., Mellemgaard A., Skov T., Skov B.G. (2009). Different Impact of Excision Repair Cross-Complementation Group 1 on Survival in Male and Female Patients with Inoperable Non-Small-Cell Lung Cancer Treated with Carboplatin and Gemcitabine. J. Clin. Oncol..

[30] Warren R.S., Atreya C.E., Niedzwiecki D., Weinberg V.K., Donner D.B., Mayer R.J., Goldberg R.M., Compton C.C., Zuraek M.B., Ye C. (2013). Association of TP53 Mutational Status and Gender with Survival after Adjuvant Treatment for Stage III Colon Cancer: Results of CALGB 89803. Clin. Cancer Res..

[31] Ning Y., Gerger A., Zhang W., Hanna D.L., Yang D., Winder T., Wakatsuki T., Labonte M.J., Stintzing S., Volz N. (2014). Plastin Polymorphisms Predict Gender- and Stage-Specific Colon Cancer Recurrence after Adjuvant Chemotherapy. Mol. Cancer Ther..

[32] Schwab M., Zanger U.M., Marx C., Schaeffeler E., Klein K., Dippon J., Kerb R., Blievernicht J., Fischer J., Hofmann U. (2008). Role of Genetic and Nongenetic Factors for Fluorouracil Treatment-Related Severe Toxicity: A Prospective Clinical Trial by the German 5-FU Toxicity Study Group. J. Clin. Oncol..

[33] Clayton J.A., Collins F.S. (2014). Policy: NIH to Balance Sex in Cell and Animal Studies. Nat. News.

[34] Leslie R., O’Donnell C.J., Johnson A.D. (2014). GRASP: Analysis of Genotype-Phenotype Results from 1390 Genome-Wide Association Studies and Corresponding Open Access Database. Bioinformatics.

[35] MacArthur J., Bowler E., Cerezo M., Gil L., Hall P., Hastings E., Junkins H., McMahon A., Milano A., Morales J. (2017). The New NHGRI-EBI Catalog of Published Genome-Wide Association Studies (GWAS Catalog). Nucleic Acids Res..

[36] Fried L.P., Borhani N.O., Enright P., Furberg C.D., Gardin J.M., Kronmal R.A., Kuller L.H., Manolio T.A., Mittelmark M.B., Newman A. (1991). The Cardiovascular Health Study: Design and Rationale. Ann. Epidemiol..

[37] Dawber T.R., Meadors G.F., Moore F.E. (1951). Epidemiological Approaches to Heart Disease: The Framingham Study. Am. J. Public Health Nations Health.

[38] Feinleib M., Kannel W.B., Garrison R.J., McNamara P.M., Castelli W.P. (1975). The Framingham Offspring Study: Design and Preliminary Data. Prev. Med..

[39] Sonnega A., Faul J.D., Ofstedal M.B., Langa K.M., Phillips J.W., Weir D.R. (2014). Cohort Profile: The Health and Retirement Study (HRS). Int. J. Epidemiol..

[40] Nazarian A., Yashin A.I., Kulminski A.M. (2019). Genome-Wide Analysis of Genetic Predisposition to Alzheimer’s Disease and Related Sex Disparities. Alzheimer’s Res. Ther..

[41] Purcell S., Neale B., Todd-Brown K., Thomas L., Ferreira M.A.R., Bender D., Maller J., Sklar P., de Bakker P.I.W., Daly M.J. (2007). PLINK: A Tool Set for Whole-Genome Association and Population-Based Linkage Analyses. Am. J. Hum. Genet..

[42] Conomos M.P., Miller M.B., Thornton T.A. (2015). Robust Inference of Population Structure for Ancestry Prediction and Correction of Stratification in the Presence of Relatedness. Genet. Epidemiol..

[43] McArdle P.F., O’Connell J.R., Pollin T.I., Baumgarten M., Shuldiner A.R., Peyser P.A., Mitchell B.D. (2007). Accounting for Relatedness in Family Based Genetic Association Studies. Hum. Hered..

[44] Bates D., Mächler M., Bolker B., Walker S. (2015). Fitting Linear Mixed-Effects Models Using Lme4. J. Stat. Softw..

[45] Nazarian A., Arbeev K.G., Kulminski A.M. (2020). The Impact of Disregarding Family Structure on Genome-Wide Association Analysis of Complex Diseases in Cohorts with Simple Pedigrees. J. Appl. Genet..

[46] Mägi R., Morris A.P. (2010). GWAMA: Software for Genome-Wide Association Meta-Analysis. BMC Bioinform..

[47] Machiela M.J., Chanock S.J. (2015). LDlink: A Web-Based Application for Exploring Population-Specific Haplotype Structure and Linking Correlated Alleles of Possible Functional Variants. Bioinformatics.

[48] Allison P.D. (1999). Comparing Logit and Probit Coefficients across Groups. Soc. Methods Res..

[49] Yoon S., Nguyen H.C.T., Yoo Y.J., Kim J., Baik B., Kim S., Kim J., Kim S., Nam D. (2018). Efficient Pathway Enrichment and Network Analysis of GWAS Summary Data Using GSA-SNP2. Nucleic Acids Res..

[50] Yang J., Lee S.H., Goddard M.E., Visscher P.M. (2011). GCTA: A Tool for Genome-Wide Complex Trait Analysis. Am. J. Hum. Genet..

[51] Bakshi A., Zhu Z., Vinkhuyzen A.A.E., Hill W.D., McRae A.F., Visscher P.M., Yang J. (2016). Fast Set-Based Association Analysis Using Summary Data from GWAS Identifies Novel Gene Loci for Human Complex Traits. Sci. Rep..

[52] Subramanian A., Tamayo P., Mootha V.K., Mukherjee S., Ebert B.L., Gillette M.A., Paulovich A., Pomeroy S.L., Golub T.R., Lander E.S. (2005). Gene Set Enrichment Analysis: A Knowledge-Based Approach for Interpreting Genome-Wide Expression Profiles. Proc. Natl. Acad. Sci. USA.

[53] Kanehisa M., Goto S. (2000). KEGG: Kyoto Encyclopedia of Genes and Genomes. Nucleic Acids Res..

[54] Nishimura D. (2001). BioCarta. Biotech Softw. Internet Rep..

[55] Schaefer C.F., Anthony K., Krupa S., Buchoff J., Day M., Hannay T., Buetow K.H. (2009). PID: The Pathway Interaction Database. Nucleic Acids Res..

[56] Fabregat A., Jupe S., Matthews L., Sidiropoulos K., Gillespie M., Garapati P., Haw R., Jassal B., Korninger F., May B. (2018). The Reactome Pathway Knowledgebase. Nucleic Acids Res..

[57] Benjamini Y., Hochberg Y. (1995). Controlling the False Discovery Rate: A Practical and Powerful Approach to Multiple Testing. J. R. Stat. Soc. Ser. B (Methodol. ).

[58] (2007). Wellcome Trust Case Control Consortium Genome-Wide Association Study of 14,000 Cases of Seven Common Diseases and 3000 Shared Controls. Nature.

[59] Luo W., Schork N.J., Marschke K.B., Ng S.-C., Hermann T.W., Zhang J., Sanders J.M., Tooker P., Malo N., Zapala M.A. (2011). Identification of Polymorphisms Associated with Hypertriglyceridemia and Prolonged Survival Induced by Bexarotene in Treating Non-Small Cell Lung Cancer. Anticancer Res..

[60] Uhlen M., Zhang C., Lee S., Sjöstedt E., Fagerberg L., Bidkhori G., Benfeitas R., Arif M., Liu Z., Edfors F. (2017). A Pathology Atlas of the Human Cancer Transcriptome. Science.

[61] Galichon P., Mesnard L., Hertig A., Stengel B., Rondeau E. (2012). Unrecognized Sequence Homologies May Confound Genome-Wide Association Studies. Nucleic Acids Res..

[62] Boraska V., Jerončić A., Colonna V., Southam L., Nyholt D.R., Rayner N.W., Perry J.R.B., Toniolo D., Albrecht E., Ang W. (2012). Genome-Wide Meta-Analysis of Common Variant Differences between Men and Women. Hum. Mol. Genet..

[63] Colussi D., Brandi G., Bazzoli F., Ricciardiello L. (2013). Molecular Pathways Involved in Colorectal Cancer: Implications for Disease Behavior and Prevention. Int. J. Mol. Sci..

[64] Markman J.L., Shiao S.L. (2015). Impact of the Immune System and Immunotherapy in Colorectal Cancer. J. Gastrointest. Oncol..

[65] Eymin B., Gazzeri S. (2010). Role of Cell Cycle Regulators in Lung Carcinogenesis. Cell Adh. Migr..

[66] Jafri M.A., Ansari S.A., Alqahtani M.H., Shay J.W. (2016). Roles of Telomeres and Telomerase in Cancer, and Advances in Telomerase-Targeted Therapies. Genome Med..

[67] Zhang W., Mao J.-H., Zhu W., Jain A.K., Liu K., Brown J.B., Karpen G.H. (2016). Centromere and Kinetochore Gene Misexpression Predicts Cancer Patient Survival and Response to Radiotherapy and Chemotherapy. Nat. Commun..

