# Supplementary material for: Genome-Wide Analysis of Sex Disparities in the Genetic Architecture of Lung and Colorectal Cancers
Source: Genes (Basel). 2021 May 1;12(5):686. doi: 10.3390/genes12050686 (PMC8147355; doi:10.3390/genes12050686)
Supplement: Supplementary file 1 [file genes-12-00686-s001.zip › genes-1167705-supplementary.pdf]

Additional File 1

Supplementary Information for:

Genome-wide Analysis of Sex Disparities in the Genetic Architecture of Lung and Colorectal Cancers

Alireza Nazarian and Alexander M. Kulminski*
Biodemography of Aging Research Unit, Social Science Research Institute, Duke University, Durham, NC, 27705, USA

* Correspondence: kulminsk@duke.edu

This file includes:

Supporting Acknowledgment text

Tables S1-S5

Figures S1-S8

Supporting Acknowledgment

This research was supported by Grants No. P01AG043352, R01AG047310, R01AG061853, R01AG065477, and R01AG070488 from the National Institute on Aging. The funders had no role in study design, data collection and analysis, decision to publish, or preparation of the manuscript. The content is solely the responsibility of the authors and does not necessarily represent the official views of the National Institutes of Health. This manuscript was prepared using limited access datasets obtained though dbGaP (accession numbers: phs000287.v5.p1 (CHS), phs000007.v28.p10 (FHS), and phs000428.v1.p2 (HRS)) and the University of Michigan. Phenotypic HRS data are available publicly and through restricted access from http://hrsonline.isr.umich.edu/index.php?p=data.

The Cardiovascular Health Study (CHS) was supported by contracts HHSN268201200036C, HHSN268200800007C, N01-HC-85079, N01-HC-85080, N01-HC-85081, N01-HC-85082, N01-HC-85083, N01-HC-85084, N01-HC-85085, N01-HC-85086, N01-HC-35129, N01 HC-15103, N01 HC-55222, N01-HC-75150, N01-HC-45133, and N01-HC-85239; grant numbers U01 HL080295 and U01 HL130014 from the National Heart, Lung, and Blood Institute (NHLBI), and R01 AG-023629 from the National Institute on Aging, with additional contribution from the National Institute of Neurological Disorders and Stroke. A full list of principal CHS investigators and institutions can be found at https://chs-nhlbi.org/pi. This manuscript was not prepared in collaboration with CHS investigators and does not necessarily reflect the opinions or views of CHS, or the NHLBI. Support for the genotyping through the CARe Study was provided by NHLBI Contract N01-HC-65226. Additional support for infrastructure was provided by HL105756 and additional genotyping among the African-American cohort was supported in part by HL085251. DNA handling and genotyping at Cedars-Sinai Medical Center was supported in part by National Center for Research Resources grant UL1RR033176, now at the National Center for Advancing Translational Technologies CTSI grant UL1TR000124; in addition to the National Institute of Diabetes and Digestive and Kidney Diseases grant DK063491 to the Southern California Diabetes Endocrinology Research Center.

The Framingham Heart Study (FHS) is conducted and supported by the National Heart, Lung, and Blood Institute (NHLBI) in collaboration with Boston University (Contract No. N01-HC-25195 and HHSN268201500001I). This manuscript was not prepared in collaboration with investigators of the FHS and does not necessarily reflect the opinions or views of the FHS, Boston University, or NHLBI. Funding for SHARe Affymetrix genotyping was provided by NHLBI Contract N02-HL-64278. SHARe Illumina genotyping was provided under an agreement between Illumina and Boston University. Funding for CARe genotyping was provided by NHLBI Contract N01-HC-65226. Funding support for the Framingham Dementia dataset was provided by NIH/NIA grant R01 AG08122. Funding support for the Framingham Inflammatory Markers was provided by NIH grants R01 HL064753, R01 HL076784 and R01 AG028321. Funding support for the Framingham C-reactive Protein dataset was provided by NIH grants R01 HL064753, R01 HL076784 and R01 AG028321. Funding support for the Framingham Adiponectin dataset was provided by NIH/NHLBI grant R01-DK-080739. Funding support for the Framingham Interleukin-6 dataset was provided by NIH grants R01 HL064753, R01 HL076784 and R01 AG028321.

The Health and Retirement Study (HRS) genetic data is sponsored by the Genetics Resource with HRS April 21, 2010, version G Page 5 of 7 National Institute on Aging (grant numbers U01AG009740, RC2AG036495, and RC4AG039029) and was conducted by the University of Michigan. This manuscript was not prepared in collaboration with HRS investigators and does not necessarily reflect the opinions or views of HRS.

Tables

Table S1. Demographic information about the analyzed datasets.

Cohort	Females					Males					Comparison		
	N	Case%	Smoke%	BY (SD)	BMI (SD)	N	Case%	Smoke%	BY (SD)	BMI (SD)	P_{Smoke}	P_{BY}	P_{BMI}
**CRCa**													
**CHS**	1943	2.47	43.39	1915.01 (5.30)	26.42 (4.90)	1282	3.82	66.77	1913.92 (5.82)	26.44 (3.63)	< 0.05	< 0.05	> 0.05
**FHS**	2809	2.60	56.89	1930.73 (15.67)	26.29 (4.91)	2276	2.86	68.76	1931.18 (14.32)	27.64 (3.75)	< 0.05	> 0.05	< 0.05
**HRS**	3841	2.34	60.77	1933.20 (8.22)	26.74 (4.99)	2940	2.45	77.45	1933.40 (7.71)	27.52 (3.95)	< 0.05	> 0.05	< 0.05
**LCa**													
**CHS**	1943	3.24	43.39	1915.01 (5.30)	26.42 (4.90)	1282	3.59	66.77	1913.92 (5.82)	26.44 (3.63)	< 0.05	< 0.05	> 0.05
**FHS**	2809	2.63	56.89	1930.73 (15.67)	26.29 (4.91)	2276	3.95	68.76	1931.18 (14.32)	27.64 (3.75)	< 0.05	> 0.05	< 0.05
**HRS**	3839	2.60	60.64	1933.23 (8.19)	26.75 (5.00)	2940	2.86	77.38	1933.42 (7.69)	27.54 (3.95)	< 0.05	> 0.05	< 0.05

Abbreviations: CRCa = colorectal cancer; LCa = lung cancer; CHS = Cardiovascular Health Study [1]; FHS = Framingham Heart Study [2,3]; HRS = Health and Retirement Study [4]; N = numbers of subjects in analyzed datasets; Case% = percent of cases; Smoke% = percent of smokers; BY (SD) = average birth year and its standard deviation; BMI (SD) = average body mass index and its standard deviation; P_{Smoke}, P_{BY}, and P_{BMI} = P-values of the differences in the distributions of covariates between the two sexes obtained by chi-square test (smoking history) or Student's t-test (birth year and BMI)

Table S2. Numbers (and percentages) of analyzed SNPs along with the genomic inflation factors (λ) resulted from our genome-wide association analyses.

Cohort	SNPs-G	SNPs-IMP	λ-G	λ-IMP
**CRCa-F**				
**HRS**	1270859	396123	1.010	1.023
**FHS**	342070	1005527	1.009	1.006
**CHS**	315263	1176540	1.023	1.023
**LCa-F**				
**HRS**	1270744	396232	1.000	1.000
**FHS**	342119	1005542	1.036	1.025
**CHS**	315308	1176805	1.007	1.008
**CRCa-M**				
**HRS**	1271669	396746	1.025	1.027
**FHS**	349661	1006039	0.999	0.993
**CHS**	315246	1176611	1.002	0.999
**LCa-F**				
**HRS**	1271601	396833	1.007	1.002
**FHS**	349752	1006061	1.006	1.011
**CHS**	314979	1176675	1.019	1.018

Abbreviations: SNP = single-nucleotide polymorphism; CRCa-F = colorectal cancer in females; LCa-F = lung cancer in females; CRCa-M = colorectal cancer in males; LCa-M = lung cancer in males; CHS = Cardiovascular Health Study [1]; FHS = Framingham Heart Study [2,3]; HRS = Health and Retirement Study [4]; SNPs-G= number of genotyped SNPs; SNPs-IMP= number of imputed SNPs; λ-G: genomic inflation factor of genotyped SNPs; λ-IMP: genomic inflation factor of imputed SNPs

Table S3. The odds ratios of fixed-effects covariates in females and males from conducted meta-analyses.

Covariate	Females			Males			Comparison	
	OR	se	P-value	OR	se	P-value	Chi-square	P-value
**CRCa**								
**Smoking history**	1.643	0.122	$4.56x10^{-5}$	1.441	0.153	$1.69x10^{-2}$	0.451	$5.02x10^{-1}$
**Birth year**	1.276	0.058	$2.73x10^{-5}$	1.185	0.067	$1.15x10^{-2}$	0.696	$4.04x10^{-1}$
**BMI**	1.027	0.010	$7.74x10^{-3}$	1.058	0.014	$8.96x10^{-5}$	2.756	$9.69x10^{-2}$
**LCa**								
**Smoking history**	4.281	0.146	$2.32x10^{-23}$	3.464	0.206	$1.74x10^{-9}$	0.702	$4.02x10^{-1}$
**Birth year**	1.473	0.059	$5.31x10^{-11}$	1.428	0.066	$6.57x10^{-8}$	0.119	$7.30x10^{-1}$
**BMI**	0.995	0.011	$6.27x10^{-1}$	0.974	0.015	$7.74x10^{-2}$	1.364	$2.43x10^{-1}$

Abbreviations: CRCa = colorectal cancer; LCa = lung cancer; BMI = body mass index; OR and se = odds ratio and its standard error

Table S4. Cancer-associated SNPs from genome-wide association analyses.

Detected Associations					HRS				FHS				CHS				Meta-analysis								Previous Findings				
**Chr**	**Gene**	**SNP**	**Pos**	**A1**	**Freq**	**OR**	**se**	P_{HRS}	**Freq**	**OR**	**se**	P_{FHS}	**Freq**	**OR**	**se**	P_{CHS}	**Freq**	**OR**	**se**	P_{META}	P_{Q}	i^2	**Effects**	**N**	**1Mb?**	**Proxy?**	**Region?**	**Gene?**	**Prognostic?**
**CRCa-F**																													
8q22.3	*SNX31*	rs1078186	100613251	C	0.579	0.582	0.153	4.19×10^{-4}	0.567	0.563	0.174	9.48×10^{-4}	0.572	0.775	0.208	2.21×10^{-1}	0.574	0.615	0.056	1.60×10^{-6}	4.49×10^{-1}	0	---	8590	N	N	S	N	O
8q24.23	*KHDRBS3*	rs118174020	135843271	C	NA	NA	NA	NA	0.949	0.370	0.281	3.92×10^{-4}	0.948	0.340	0.324	8.73×10^{-4}	0.949	0.357	0.062	1.41×10^{-6}	8.49×10^{-1}	0	?--	4752	N	N	N	O	O
10q26.3	*GLRX3*	rs3118492	130435775	A	0.227	1.624	0.162	2.74×10^{-3}	0.232	1.711	0.203	8.05×10^{-3}	0.230	1.720	0.227	1.69×10^{-2}	0.229	1.672	0.168	4.15×10^{-6}	9.71×10^{-1}	0	+++	8544	N	N	N	N	O
10q26.3	*GLRX3*	rs372813	130442933	A	0.227	1.619	0.162	2.89×10^{-3}	0.243	1.678	0.189	6.08×10^{-3}	0.230	1.721	0.227	1.68×10^{-2}	0.233	1.661	0.163	3.25×10^{-6}	9.75×10^{-1}	0	+++	8592	N	N	N	N	O
10q26.3	*GLRX3*	rs359063	130452326	T	0.225	1.585	0.163	4.64×10^{-3}	0.239	1.732	0.190	3.89×10^{-3}	0.227	1.751	0.228	1.39×10^{-2}	0.230	1.669	0.164	2.86×10^{-6}	9.14×10^{-1}	0	+++	8545	N	N	N	N	O
10q26.3	*GLRX3*	rs307385	130452402	T	0.225	1.604	0.162	3.61×10^{-3}	0.238	1.754	0.192	3.50×10^{-3}	0.227	1.751	0.228	1.39×10^{-2}	0.230	1.684	0.167	2.16×10^{-6}	9.23×10^{-1}	0	+++	8543	N	N	N	N	O
**LCa-F**																													
2p21	*LOC102723824*	rs7593032*	42122050	T	0.712	0.506	0.149	4.63×10^{-6}	0.709	0.841	0.179	3.32×10^{-1}	0.719	0.663	0.197	3.68×10^{-2}	0.713	0.631	0.057	3.66×10^{-6}	9.14×10^{-2}	0.582	---	8577	N	N	S	N	N
5q13.2	*LINC02056*	rs42775	72584687	T	0.329	1.701	0.145	2.43×10^{-4}	0.328	1.527	0.181	1.97×10^{-2}	0.315	1.381	0.188	8.54×10^{-2}	0.325	1.561	0.138	4.98×10^{-6}	6.74×10^{-1}	0	+++	8516	S	N	S	N	N
10q21.1	*PRKG1*	rs11000463*	52014359	T	0.897	0.427	0.186	4.60×10^{-6}	0.908	0.588	0.255	3.77×10^{-2}	0.906	1.097	0.311	7.65×10^{-1}	0.903	0.558	0.067	1.75×10^{-5}	3.31×10^{-2}	0.707	--+	8591	N	N	N	O	N
10q21.1	*PRKG1*	rs11000467*	52015312	G	0.897	0.427	0.186	4.67×10^{-6}	0.914	0.552	0.272	2.87×10^{-2}	0.906	1.097	0.311	7.65×10^{-1}	0.905	0.548	0.066	1.37×10^{-5}	3.41×10^{-2}	0.704	--+	8570	N	N	N	O	N
10q26.13	*ZRANB1*	rs76972397	124961380	T	0.925	0.455	0.210	1.70×10^{-4}	NA	NA	NA	NA	0.938	0.390	0.295	1.43×10^{-3}	0.930	0.432	0.063	9.62×10^{-7}	6.72×10^{-1}	0	-?-	5770	N	N	G	O	N
18q11.2	*HRH4*	rs482962	24532843	A	0.272	1.408	0.152	2.41×10^{-2}	0.237	1.871	0.203	2.04×10^{-3}	0.277	1.747	0.196	4.52×10^{-3}	0.262	1.607	0.151	4.90×10^{-6}	4.75×10^{-1}	0	+++	8585	N	N	S	N	N
**CRCa-M**																													
3p24.2	*THRB*	rs57751578	24486090	T	0.902	0.459	0.224	5.16×10^{-4}	0.919	0.587	0.281	5.80×10^{-2}	0.917	0.423	0.302	4.39×10^{-3}	0.911	0.483	0.064	1.89×10^{-6}	6.98×10^{-1}	0	---	6491	N	N	N	O	O
5q31.1	*FSTL4*	rs4631227	133152748	A	0.284	1.914	0.173	1.81×10^{-4}	0.299	1.410	0.181	5.77×10^{-2}	0.303	1.702	0.218	1.49×10^{-2}	0.293	1.663	0.163	3.20×10^{-6}	4.76×10^{-1}	0	+++	6481	N	N	G	O	N
6q14.3	*HTR1E*	rs72907251	86515650	C	0.933	0.386	0.240	7.49×10^{-5}	0.942	0.425	0.270	1.55×10^{-3}	0.941	0.743	0.405	4.64×10^{-1}	0.937	0.446	0.063	1.06×10^{-6}	3.73×10^{-1}	0	---	6496	N	N	N	O	N
13q12.11	*MPHOSPH8*	rs9579517*	19603453	C	0.902	0.375	0.208	2.43×10^{-6}	0.895	0.961	0.292	8.91×10^{-1}	0.904	0.523	0.291	2.62×10^{-2}	0.900	0.519	0.066	8.88×10^{-6}	3.31×10^{-2}	0.707	---	6490	N	N	N	N	N
13q12.11	*MPHOSPH8*	rs56357430*	19632421	C	0.902	0.375	0.208	2.43×10^{-6}	0.897	1.031	0.301	9.20×10^{-1}	0.900	0.544	0.292	3.69×10^{-2}	0.900	0.528	0.068	1.77×10^{-5}	2.25×10^{-2}	0.736	-+-	6498	N	N	N	N	N
**LCa-M**																													
5p15.33	*LINC01377*	rs12657742	3123661	A	0.869	0.577	0.200	5.98×10^{-3}	0.857	0.547	0.201	2.66×10^{-3}	0.867	0.527	0.281	2.27×10^{-2}	0.864	0.555	0.062	3.67×10^{-6}	9.63×10^{-1}	0	---	6490	N	N	G	O	N
5q23.2	*LINC02039*	rs77914729	126003571	C	0.925	0.528	0.233	6.07×10^{-3}	0.933	0.418	0.272	1.33×10^{-3}	0.931	0.464	0.346	2.67×10^{-2}	0.929	0.475	0.065	2.67×10^{-6}	8.09×10^{-1}	0	---	6495	N	N	S	N	N
5q23.2	*LINC02039*	rs75874914	126003878	C	0.925	0.529	0.233	6.25×10^{-3}	0.933	0.425	0.271	1.64×10^{-3}	0.932	0.464	0.346	2.66×10^{-2}	0.929	0.478	0.065	3.24×10^{-6}	8.29×10^{-1}	0	---	6496	N	N	S	N	N
5q23.2	*LINC02039*	rs11954381	126006435	G	0.926	0.521	0.234	5.32×10^{-3}	0.933	0.430	0.269	1.73×10^{-3}	0.932	0.464	0.346	2.66×10^{-2}	0.929	0.477	0.065	2.81×10^{-6}	8.63×10^{-1}	0	---	6491	N	N	S	N	N
6q23.3	*MAP7*	rs9399183	136496350	A	0.832	0.468	0.172	1.03×10^{-5}	0.839	0.711	0.192	7.56×10^{-2}	0.823	0.679	0.258	1.33×10^{-1}	0.833	0.585	0.060	3.33×10^{-6}	2.19×10^{-1}	0.341	---	6494	N	N	N	N	O
6q23.3	*MAP7*	rs3799451	136508794	A	0.851	0.448	0.176	4.91×10^{-6}	0.860	0.684	0.201	5.78×10^{-2}	0.842	0.691	0.263	1.61×10^{-1}	0.852	0.565	0.060	1.62×10^{-6}	2.01×10^{-1}	0.377	---	6495	N	N	N	N	O
6q23.3	*MAP7*	rs3799453	136518050	G	0.852	0.447	0.176	4.70×10^{-6}	0.860	0.684	0.201	5.78×10^{-2}	0.843	0.728	0.271	2.42×10^{-1}	0.853	0.570	0.061	2.46×10^{-6}	1.72×10^{-1}	0.432	---	6495	N	N	N	N	O
6q23.3	*MAP7*	rs3799454	136518078	T	0.835	0.462	0.172	6.90×10^{-6}	0.841	0.706	0.191	6.85×10^{-2}	0.824	0.724	0.261	2.16×10^{-1}	0.835	0.586	0.060	3.58×10^{-6}	1.76×10^{-1}	0.425	---	6498	N	N	N	N	O
6q23.3	*MAP7*	rs3799462	136524611	G	0.852	0.459	0.177	1.10×10^{-5}	0.860	0.684	0.201	5.78×10^{-2}	0.843	0.728	0.271	2.42×10^{-1}	0.853	0.577	0.062	4.49×10^{-6}	2.12×10^{-1}	0.356	---	6498	N	N	N	N	O
8p23.2	*CSMD1*	rs13261356	4605119	C	0.904	0.502	0.210	1.04×10^{-3}	0.895	0.458	0.233	8.05×10^{-4}	NA	NA	NA	NA	0.900	0.482	0.065	3.23×10^{-6}	7.69×10^{-1}	0	--?	5209	G	N	G	SO	N
10p12.31	*MIR4675*	rs11012129	20545511	T	0.947	0.461	0.253	2.15×10^{-3}	0.942	0.284	0.292	1.68×10^{-5}	NA	NA	NA	NA	0.945	0.375	0.060	3.21×10^{-7}	2.14×10^{-1}	0.352	--?	5207	N	N	S	O	N
18q21.31	*ATP8B1*	rs2437037	57795758	A	0.909	0.458	0.213	2.39×10^{-4}	NA	NA	NA	NA	0.910	0.432	0.296	4.50×10^{-3}	0.909	0.449	0.066	3.95×10^{-6}	8.71×10^{-1}	0	-?-	4216	N	N	N	N	O
21q21.3	*GRIK1*	rs363433	29588811	C	0.855	0.535	0.187	8.08×10^{-4}	0.865	0.571	0.219	1.03×10^{-2}	0.876	0.499	0.277	1.20×10^{-2}	0.863	0.539	0.061	1.14×10^{-6}	9.30×10^{-1}	0	---	6490	N	N	N	O	O
21q21.3	*GRIK1*	rs363432	29591270	G	0.857	0.570	0.191	3.23×10^{-3}	0.862	0.573	0.217	1.03×10^{-2}	0.877	0.496	0.277	1.12×10^{-2}	0.863	0.554	0.063	4.10×10^{-6}	9.03×10^{-1}	0	---	6495	N	N	N	O	O
21q21.3	*GRIK1*	rs2832405	29591425	T	0.849	0.530	0.184	5.58×10^{-4}	0.852	0.589	0.211	1.24×10^{-2}	0.871	0.523	0.278	1.98×10^{-2}	0.854	0.548	0.061	1.50×10^{-6}	9.16×10^{-1}	0	---	6497	N	N	N	O	O
21q21.3	*GRIK1*	rs363472	29595793	G	0.852	0.559	0.186	1.77×10^{-3}	0.852	0.594	0.210	1.29×10^{-2}	0.872	0.518	0.278	1.78×10^{-2}	0.856	0.562	0.062	4.23×10^{-6}	9.26×10^{-1}	0	---	6490	N	N	N	O	O

Abbreviations: SNP = single-nucleotide polymorphism; CRCa-F = colorectal cancer in females; LCa-F = lung cancer in females; CRCa-M = colorectal cancer in males; LCa-M = lung cancer in males; CHS = Cardiovascular Health Study [1]; FHS = Framingham Heart Study [2,3]; HRS = Health and Retirement Study [4]; Chr = chromosomal region (i.e., cytogenetic band); Gene = the gene within which the SNP is located or else the closest gene to the SNP; Pos = SNP position based on Human Genome version 38 (hg38); A1 = effect allele; Freq = frequency of effect allele; OR (se) = odds ratio and its standard error; P_{HRS}, P_{FHS}, and P_{CHS} = P-values of the SNP in HRS, FHS, and CHS datasets, respectively; P_{META} = P-value in conducted meta-analysis; P_{Q} = P-value of Q-statistics (Cochran's heterogeneity test); i^2 = i-squared inconsistency metric; Effects = directions of SNP's effects in HRS, FHS, and CHS datasets, respectively (Positive, Negative, Missing); N = Number of subjects with non-missing genotype data in conducted meta-analysis; 1Mb? = whether any polymorphism associated with the same cancer was previously discovered within the ±1 Mb of the SNP detected here (G: polymorphism with $P < 5.0 \times 10^{-8}$, and S: polymorphism with $5.0 \times 10^{-8} \le P < 5.0 \times 10^{-6}$, N: none); Proxy? = whether any of the 1Mb?-SNPs is in linkage disequilibrium (LD) with the SNP detected here and its P-value is less than the one detected in this study (Y: yes, N: no); Region? = whether any SNP within the chromosomal region corresponding to the SNP detected here was previously associated with the same cancer at $P < 5.0 \times 10^{-6}$ (Y: yes, N: no); Gene? = whether any SNP mapped to the gene corresponding to the SNP detected here was previously associated with cancers at $P < 5.0 \times 10^{-6}$ (S: same cancer, O: other cancers, N: none); Prognostic? = whether the closest gene to the SNP detected here was previously reported as a cancer prognostic factor at $P < 0.001$ (S: same cancer, O: other cancers, N: none) [The Human Protein Atlas [5]: www.proteinatlas.org]; * = Replicated SNPs

Table S5. Linkage disequilibrium measures among SNPs with significant association signals that were mapped to the same gene.

SNP1	SNP2	D'	r^2
GLRX3			
rs3118492	rs372813	1	1
rs3118492	rs359063	1	0.971
rs3118492	rs307385	1	0.971
rs372813	rs359063	1	0.971
rs372813	rs307385	1	0.971
rs359063	rs307385	1	1
PRKG1			
rs11000463	rs11000467	1	1
MPHOSPH8			
rs9579517	rs56357430	1	1
LINC02039			
rs77914729	rs75874914	1	1
rs77914729	rs11954381	1	1
rs75874914	rs11954381	1	1
MAP7			
rs9399183	rs3799451	1	0.855
rs9399183	rs3799453	1	0.855
rs9399183	rs3799454	1	1
rs9399183	rs3799462	1	0.855
rs3799451	rs3799453	1	1
rs3799451	rs3799454	1	0.855
rs3799451	rs3799462	1	1
rs3799453	rs3799454	1	0.855
rs3799453	rs3799462	1	1
rs3799454	rs3799462	1	0.855
GRIK1			
rs363433	rs363432	1	1
rs363433	rs2832405	1	0.957
rs363433	rs363472	1	0.957
rs363432	rs2832405	1	0.957
rs363432	rs363472	1	0.957
rs2832405	rs363472	1	1

The LD measures were obtained for the CEU population (i.e., Utah Residents with Northern and Western European Ancestry) using *LDlink* web-tool [6].

Figures

Figure S1. Manhattan plot of the genome-wide association analyses of colorectal cancer in females (CRCa-F).

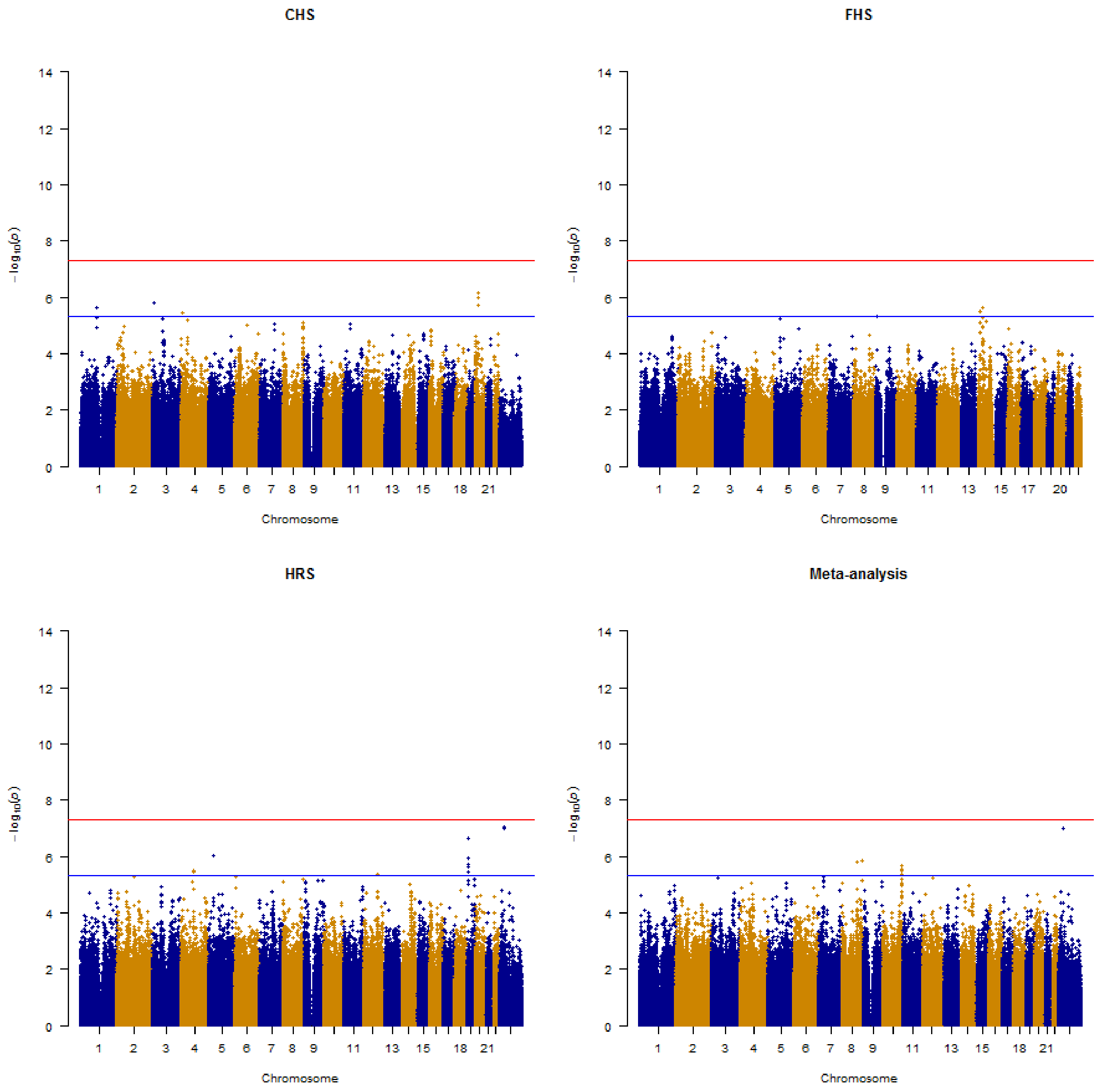

Abbreviations: CHS = Cardiovascular Health Study [1]; FHS = Framingham Heart Study [2,3]; HRS = Health and Retirement Study [4]; Red and blue lines display the genome-wide (i.e., $P < 5.0 \times 10^{-8}$) and suggestive (i.e., $5.0 \times 10^{-8} \leq P < 5.0 \times 10^{-6}$) significance levels, respectively.

Figure S2. QQ plot of the genome-wide association analyses of colorectal cancer in females (CRCa-F).

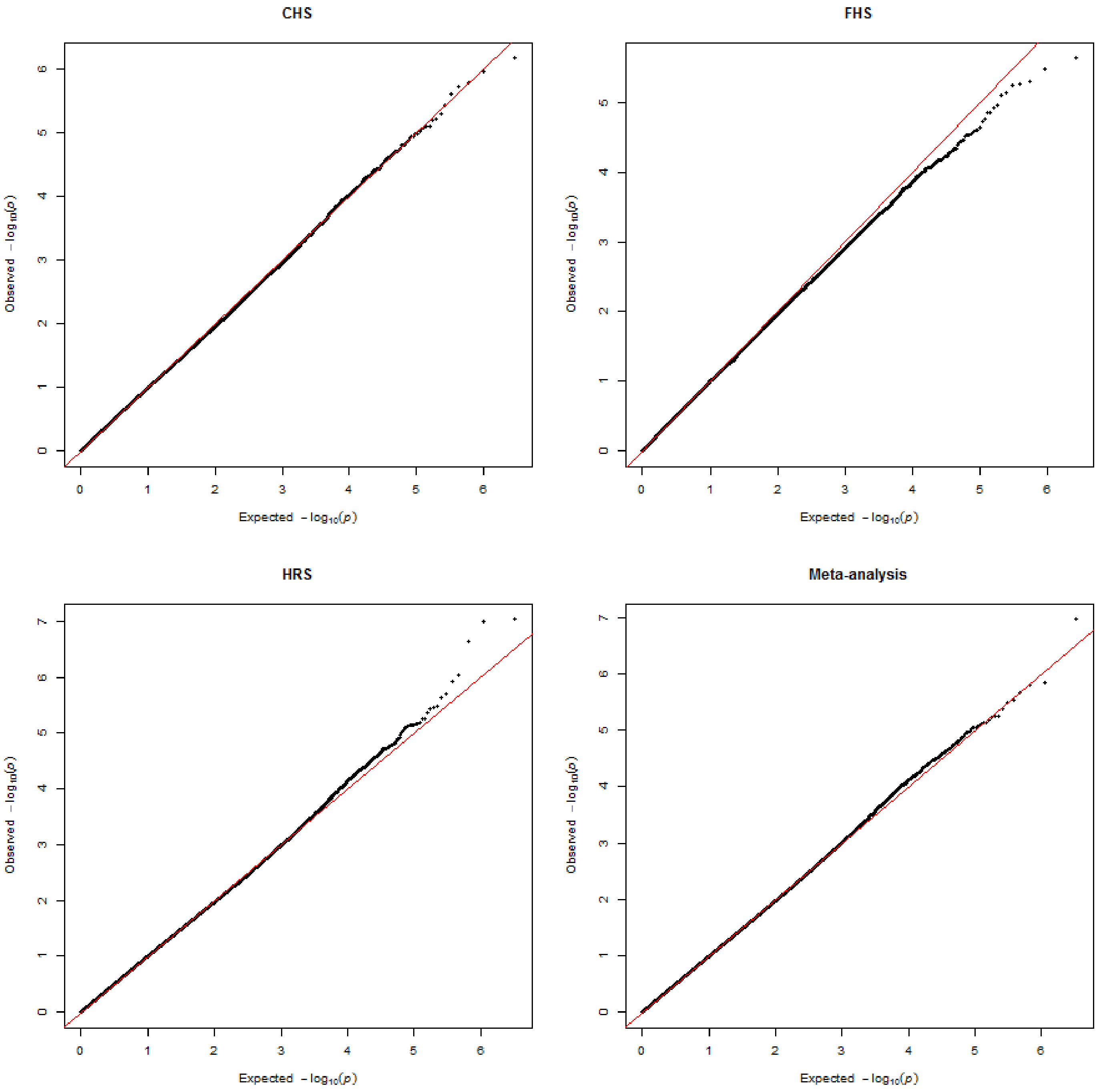

Abbreviations: CHS = Cardiovascular Health Study [1]; FHS = Framingham Heart Study [2,3]; HRS = Health and Retirement Study [4]

Figure S3. Manhattan plot of the genome-wide association analyses of lung cancer in females (LCa-F).

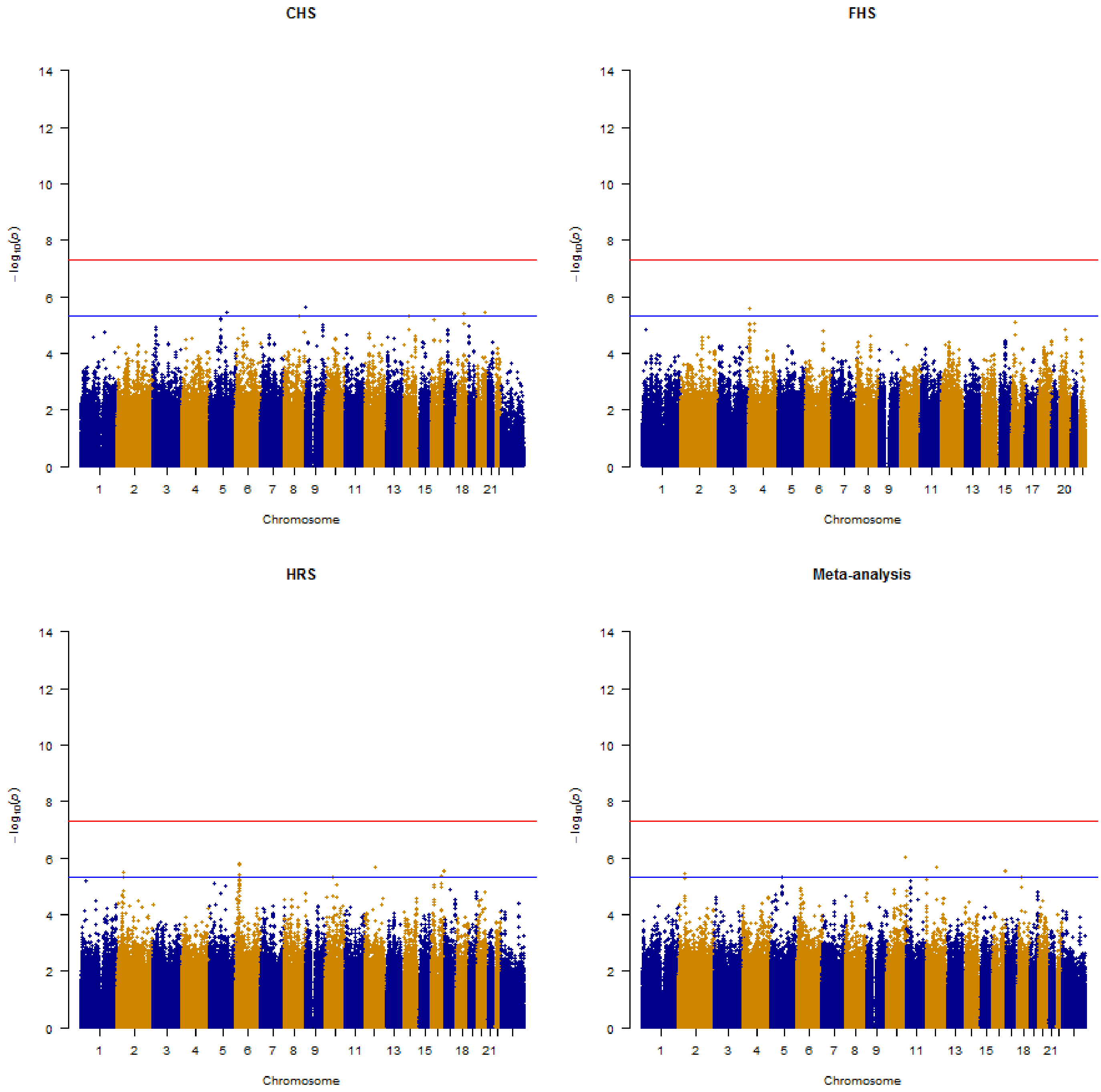

Abbreviations: CHS = Cardiovascular Health Study [1]; FHS = Framingham Heart Study [2,3]; HRS = Health and Retirement Study [4]; Red and blue lines display the genome-wide (i.e., $P < 5.0 \times 10^{-8}$) and suggestive (i.e., $5.0 \times 10^{-8} \leq P < 5.0 \times 10^{-6}$) significance levels, respectively.

Figure S4. QQ plot of the genome-wide association analyses of lung cancer in females (LCa-F).

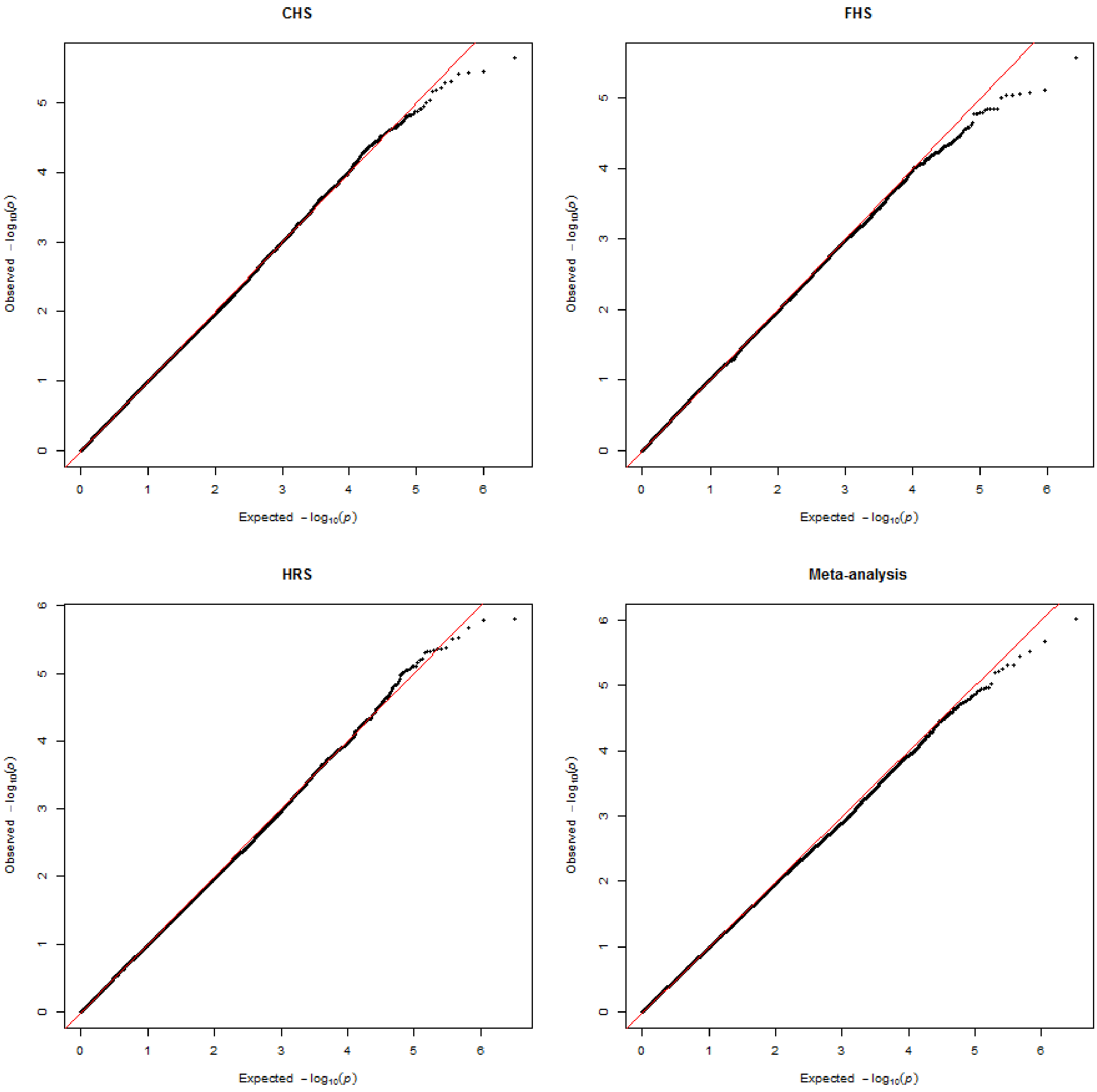

Abbreviations: CHS = Cardiovascular Health Study [1]; FHS = Framingham Heart Study [2,3]; HRS = Health and Retirement Study [4]

Figure S5. Manhattan plot of the genome-wide association analyses of colorectal cancer in males (CRCa-M).

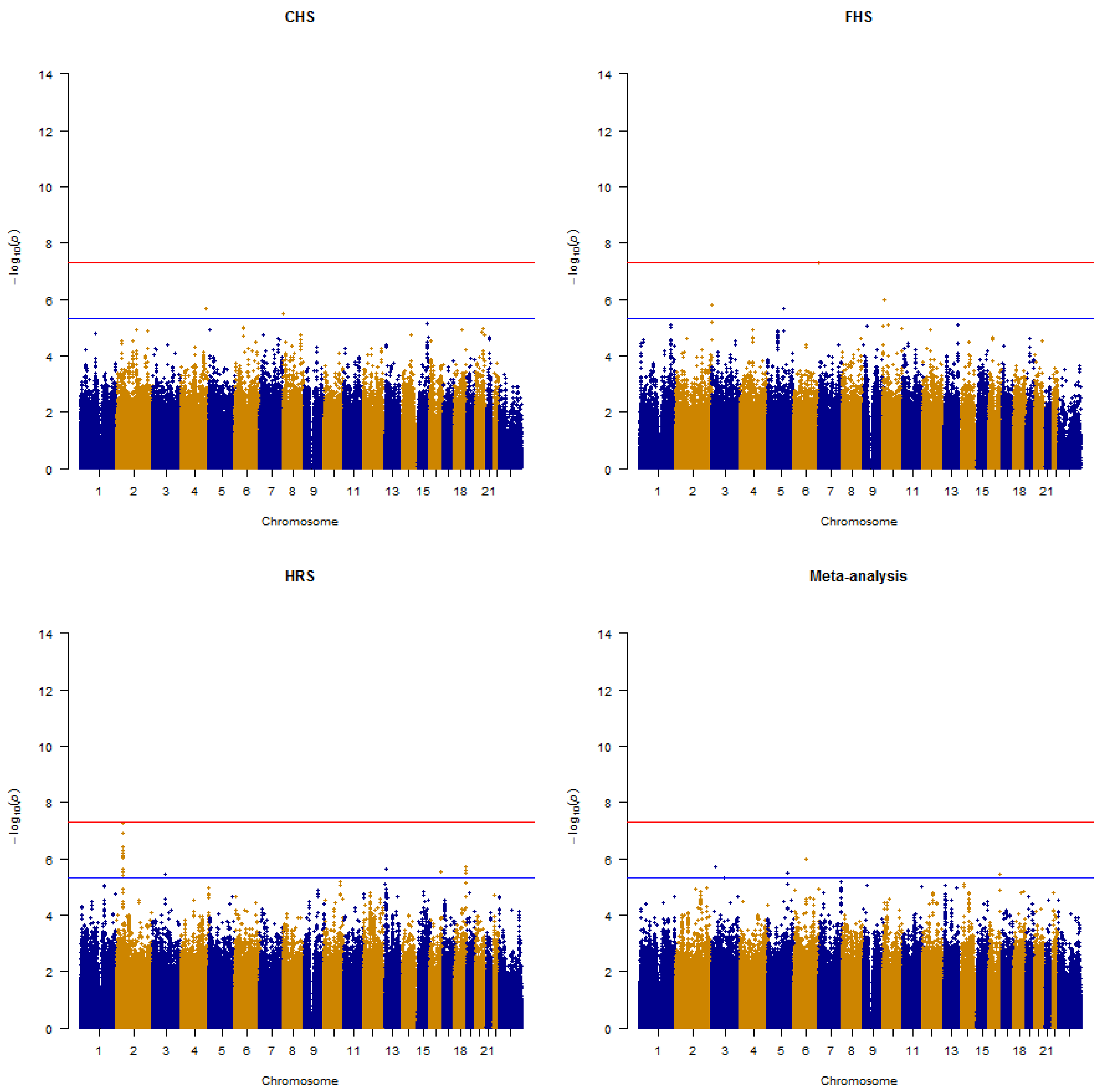

Abbreviations: CHS = Cardiovascular Health Study [1]; FHS = Framingham Heart Study [2,3]; HRS = Health and Retirement Study [4]; Red and blue lines display the genome-wide (i.e., $P < 5.0 \times 10^{-8}$) and suggestive (i.e., $5.0 \times 10^{-8} \leq P < 5.0 \times 10^{-6}$) significance levels, respectively.

Figure S6. QQ plot of the genome-wide association analyses of colorectal cancer in males (CRCa-M).

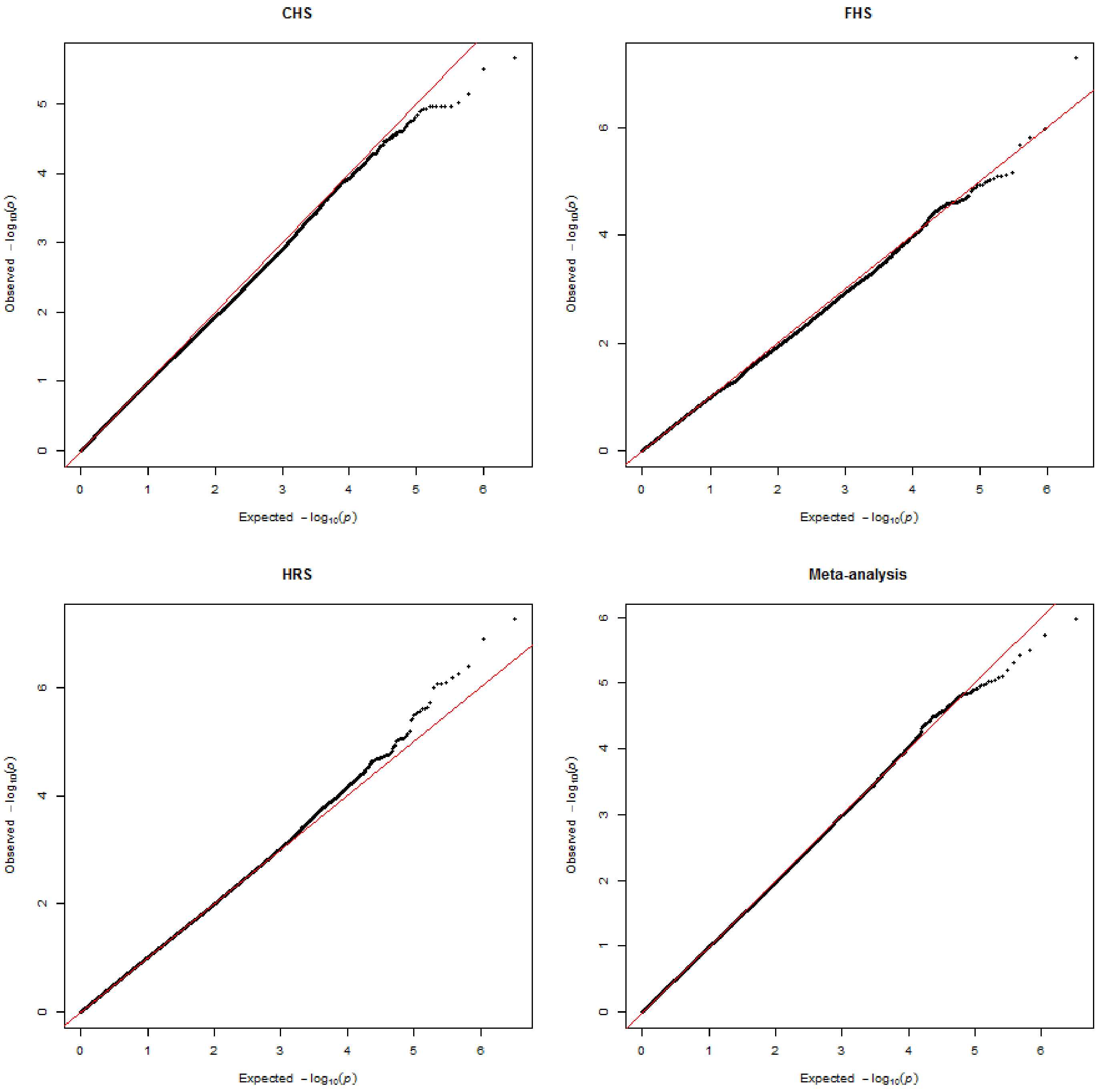

Abbreviations: CHS = Cardiovascular Health Study [1]; FHS = Framingham Heart Study [2,3]; HRS = Health and Retirement Study [4]

Figure S7. Manhattan plot of the genome-wide association analyses of lung cancer in males (LCa-M).

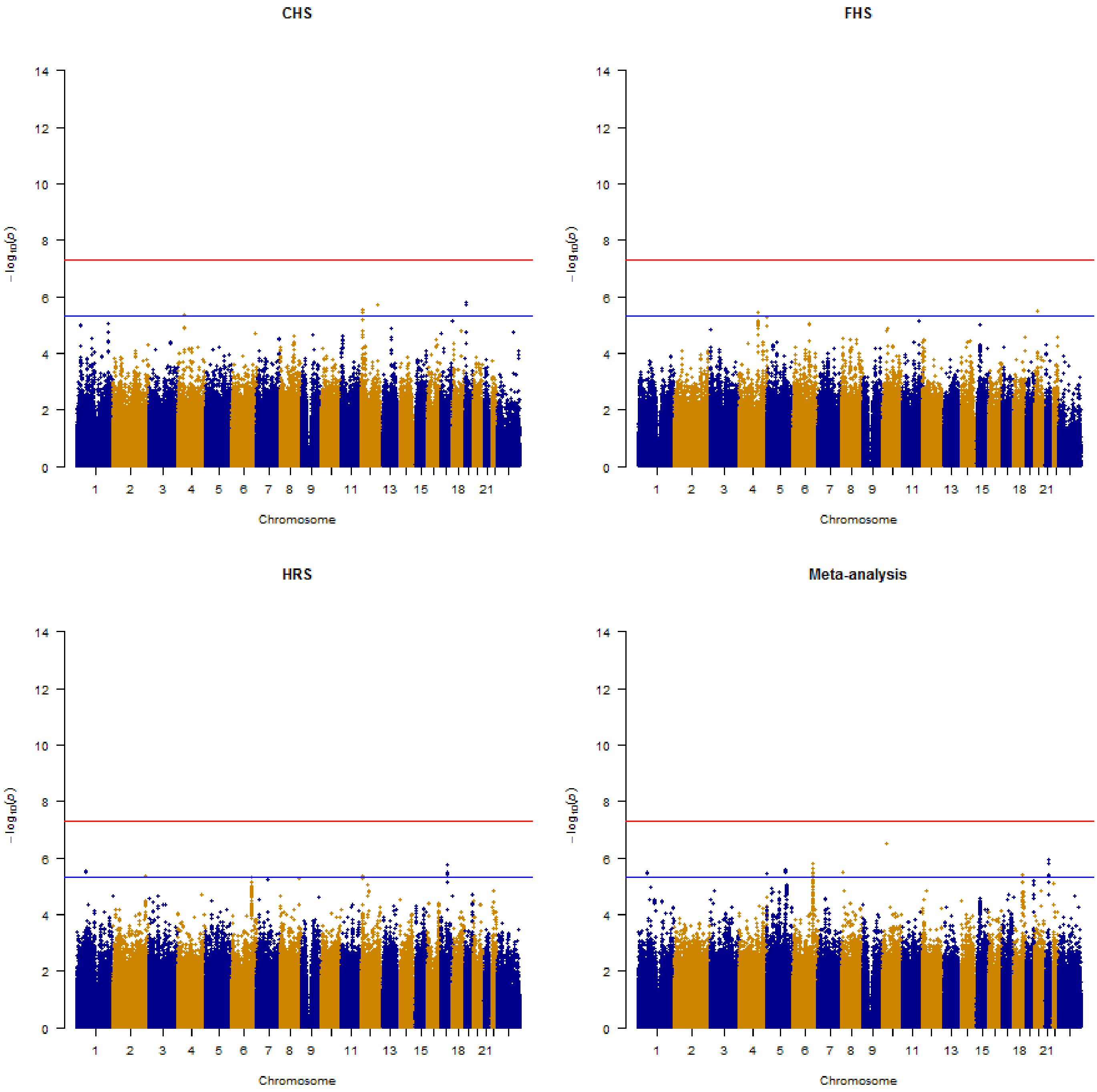

Abbreviations: CHS = Cardiovascular Health Study [1]; FHS = Framingham Heart Study [2,3]; HRS = Health and Retirement Study [4]; Red and blue lines display the genome-wide (i.e., $P < 5.0 \times 10^{-8}$) and suggestive (i.e., $5.0 \times 10^{-8} \leq P < 5.0 \times 10^{-6}$) significance levels, respectively.

Figure S8. QQ plot of the genome-wide association analyses of lung cancer in males (LCa-M).

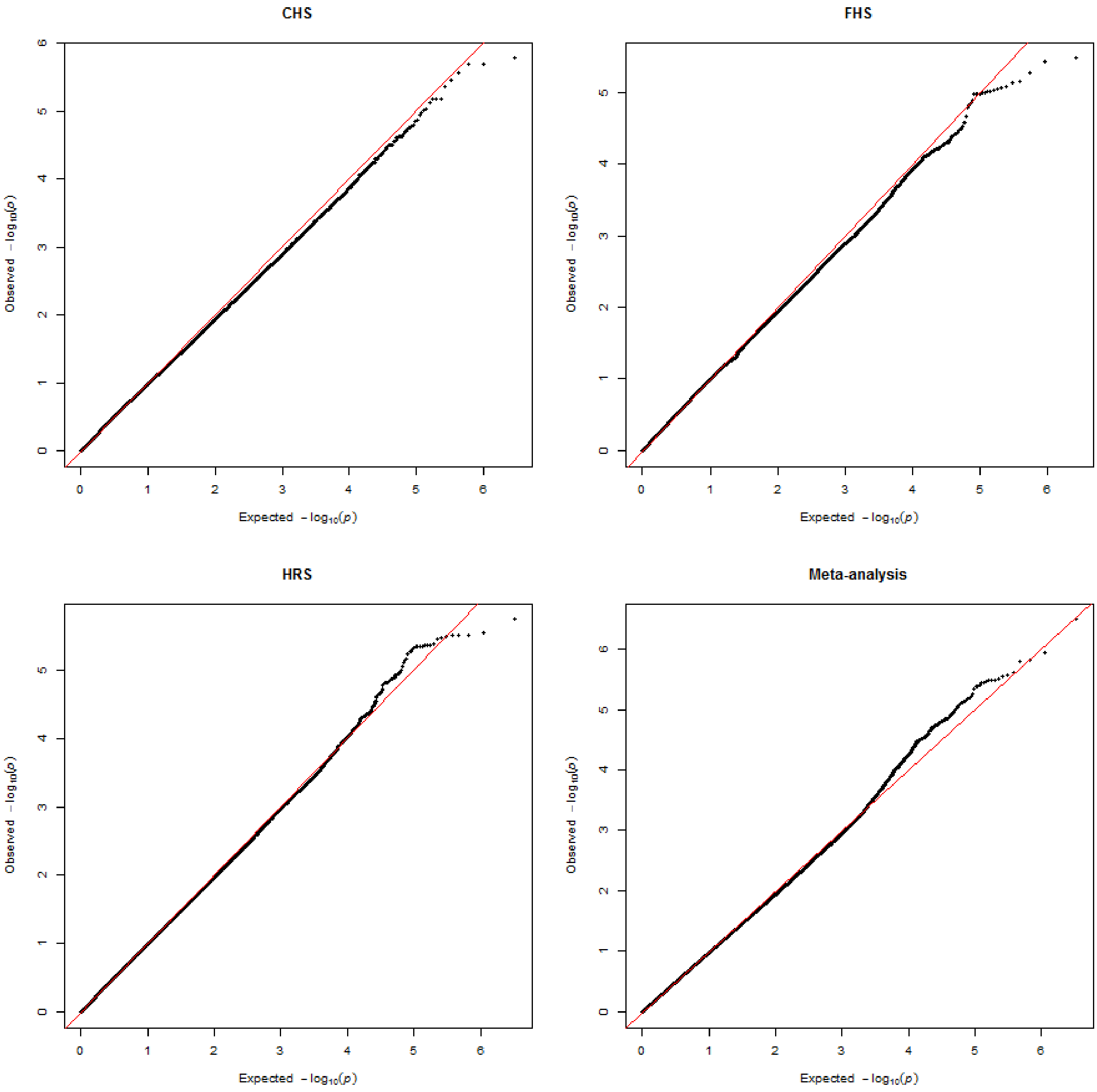

Abbreviations: CHS = Cardiovascular Health Study [1]; FHS = Framingham Heart Study [2,3]; HRS = Health and Retirement Study [4]

References

1. Fried, L.P.; Borhani, N.O.; Enright, P.; Furberg, C.D.; Gardin, J.M.; Kronmal, R.A.; Kuller, L.H.; Manolio, T.A.; Mittelmark, M.B.; Newman, A. The Cardiovascular Health Study: Design and Rationale. *Ann Epidemiol* **1991**, *1*, 263–276, doi:10.1016/1047-2797(91)90005-W.
2. Dawber, T.R.; Meadors, G.F.; Moore, F.E. Epidemiological Approaches to Heart Disease: The Framingham Study. *Am J Public Health Nations Health* **1951**, *41*, 279–286, doi:10.2105/ajph.41.3.279.
3. Feinleib, M.; Kannel, W.B.; Garrison, R.J.; McNamara, P.M.; Castelli, W.P. The Framingham Offspring Study: Design and Preliminary Data. *Prev Med* **1975**, *4*, 518–525, doi:10.1016/0091-7435(75)90037-7.
4. Sonnega, A.; Faul, J.D.; Ofstedal, M.B.; Langa, K.M.; Phillips, J.W.; Weir, D.R. Cohort Profile: The Health and Retirement Study (HRS). *Int J Epidemiol* **2014**, *43*, 576–585, doi:10.1093/ije/dyu067.
5. Uhlen, M.; Zhang, C.; Lee, S.; Sjöstedt, E.; Fagerberg, L.; Bidkhori, G.; Benfeitas, R.; Arif, M.; Liu, Z.; Edfors, F.; et al. A Pathology Atlas of the Human Cancer Transcriptome. *Science* **2017**, *357*, 2507, doi:10.1126/science.aan2507.
6. Machiela, M.J.; Chanock, S.J. LDlink: A Web-Based Application for Exploring Population-Specific Haplotype Structure and Linking Correlated Alleles of Possible Functional Variants. *Bioinformatics* **2015**, *31*, 3555–3557, doi:10.1093/bioinformatics/btv402.
